# Tunable quantum gaps to decouple carrier and phonon transport leading to high-performance thermoelectrics

**DOI:** 10.1038/s41467-022-33330-9

**Published:** 2022-09-24

**Authors:** Yong Yu, Xiao Xu, Yan Wang, Baohai Jia, Shan Huang, Xiaobin Qiang, Bin Zhu, Peijian Lin, Binbin Jiang, Shixuan Liu, Xia Qi, Kefan Pan, Di Wu, Haizhou Lu, Michel Bosman, Stephen J. Pennycook, Lin Xie, Jiaqing He

**Affiliations:** 1 Shenzhen Key Laboratory of Thermoelectric Materials, Department of Physics, Southern University of Science and Technology, 518055 Shenzhen, China; 2 Department of Materials Science and Engineering, National University of Singapore, 117575 Singapore, Singapore; 3 School of Materials Science and Engineering, Shaanxi Normal University; Key Laboratory for Macromolecular Science of Shaanxi Province, 710062 Xi’an, China; 4 Guangdong-Hong Kong-Macao Joint Laboratory for Photonic-Thermal-Electrical Energy Materials and Devices, Southern University of Science and Technology, 518055 Shenzhen, China

**Keywords:** Thermoelectric devices and materials, Imaging techniques

## Abstract

Thermoelectrics enable direct heat-to-electricity transformation, but their performance has so far been restricted by the closely coupled carrier and phonon transport. Here, we demonstrate that the quantum gaps, a class of planar defects characterized by nano-sized potential wells, can decouple carrier and phonon transport by selectively scattering phonons while allowing carriers to pass effectively. We choose the van der Waals gap in GeTe-based materials as a representative example of the quantum gap to illustrate the decoupling mechanism. The nano-sized potential well of the quantum gap in GeTe-based materials is directly visualized by in situ electron holography. Moreover, a more diffused distribution of quantum gaps results in further reduction of lattice thermal conductivity, which leads to a peak *ZT* of 2.6 at 673 K and an average ZT of 1.6 (323–723 K) in a GeTe system. The quantum gap can also be engineered into other thermoelectrics, which provides a general method for boosting their thermoelectric performance.

## Abstract (web summary)

Defects are believed to always scatter carriers. Here, the authors find that the quantum gaps in GeTe-based materials do not scatter carriers, which decouple the carriers and phonons transport leading to high thermoelectric performance.

## Introduction

The work process is always accompanied by undesired but unavoidable heat dissipation^1,2^. This energy lost by heat may account for more than half of the total energy input^3,4^. It will be of great economic and environmental benefit even if a fraction of this waste heat can be collected and reused^5^. Thermoelectrics offer such a direct and environmentally friendly way to convert thermal energy to electricity. However, their applications are limited by the relatively low conversion efficiency. The figure of merit, *ZT*, is the essential measure of thermoelectric performance and can be calculated by *ZT* = *S*^2^*σT/κ*, where *S, σ, κ*, and *T* are Seebeck coefficient, electrical conductivity, total thermal conductivity and absolute temperature, respectively^4,6–10^. The power factor (*S*^2^σ, *PF*) can be boosted by energy-band engineering including band flattening^11^, density of states (DOS) distortion^12^, and band convergence^13^. The total thermal conductivity, *κ*, can be suppressed by the introduction of all scale defects from zero dimension to three dimension^14^. Although established strategies to optimize *ZT* usually treat electrical and thermal properties separately, enhancing *ZT* requires simultaneous optimization of the adversely interdependent *S*, *σ*, and *κ*^6^, which is challenging because most crystal imperfections are believed to scatter both phonons and carriers^7^.

Attempts to overcome such difficulty have been made in specific material systems. In caged materials, the transport of electrons and phonons is through different sublattices, allowing the decoupling of mobility and lattice thermal conductivity (*κ*_lattice_). Unfortunately, this strategy cannot be readily applied to other thermoelectrics with different structures^7^. More recently, the engineering of selectively scattering interfaces has garnered increasing attention since interfaces can be introduced into the matrix regardless of the crystal structure. For example, coherent and strained interfaces along with aligned valence bands have been constructed between nanosized SrTe and a PbTe matrix so that the *κ*_lattice_ is decreased while the mobility remains intact^15^. Nevertheless, the multiple features (coherent and strained interface, aligned valence band) of the second phase and the matrix make this route hardly applicable to other thermoelectrics. Thus, a kind of selectively scattering defect/interface that can be applied to many material systems is desperately sought by the thermoelectric community. However, the main obstacle is the limitation of the well-accepted classical theory of semiconductors in which defect/interface always scatters both carriers and phonons. In this work, we find that a planar defect characterized by nano-sized potential well would allow near-perfect transmission of carriers and decouple carrier and phonon transport. The quantum mechanical wave nature of carriers leads to near-perfect transmission, thus a new concept, quantum gap (QG), is proposed to describe those defects.

**Fig. 1:**
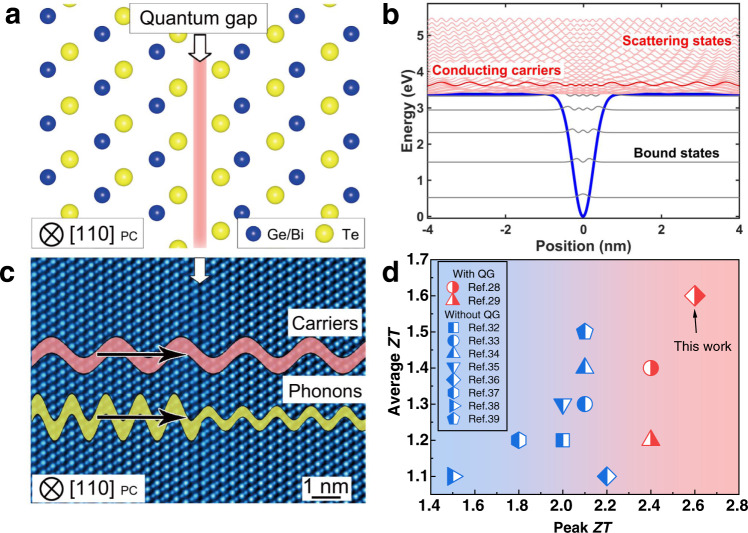
Quantum gaps and their role in improving the performance of Ge–Bi–Te alloys. **a** The structural model of a QG in Ge–Bi–Te alloy viewed along [110]_PC_ zone axis. **b** The experimental quantum well and calculated quantum states at the QG area. **c** A sketch of the transport property of QG, which allows carriers to freely pass through but strongly scatter phonons. The background is a high-angle annular dark-field scanning transmission electron microscopy (HAADF-STEM) image taken from [110]_PC_ in false color. **d** Peak *ZT*s and average *ZT*s (323–723 K) of representative high-performance lead-free GeTe thermoelectrics.

We find the van der Waals gaps (vdW gaps) in GeTe-based thermoelectrics^16–25^ is a representative example of QGs to decoupling carriers and phonons transport. It should be noted that the decoupling role of vdW gaps in GeTe-based thermoelectrics was not realized by previous works, which simply treated the vdW gaps as stacking faults to scatter phonons but did not mention whether these vdW gaps scatter charge carriers.^26–30^ The structure of a vdW gap (mentioned as QG in this work) in Ge–Bi–Te alloy is shown along the zone axis of [110]_PC_ in Fig. 1a. Here PC denotes the crystallographic index of rhombohedral material in pseudo-cubic notations. This QG would result in an atomically thin Gaussian-shaped quantum well *V*(x), in which x stands for the position (Fig. 1b). The quantum well generates quantum states with energy eigenvalues (*E*). Those *E* > either *V*(−∞) or *V*(+∞) are continuous and called scattering states, while those *E* < both *V*(−∞) and *V*(+∞) are isolated and called bound states^31^. The conducting carriers with Fermi energy are in certain scattering states, one of which is high-lighted schematically by the deep red color (Fig. 1b). Along the direction perpendicular to the QG, the carriers can perfectly transmit but the phonons are strongly scattered (Fig. 1c). The peak *ZT* and average *ZT* of our best materials together with recently reported high-performance lead-free GeTe-based polycrystal thermoelectrics are shown in Fig. 1d.^28,29,32–39^ We found that the group with QG has a more desirable peak *ZT* and *ZT*_average_ (323–723 K) compared to those without QG. Our study here further improves the peak *ZT* to 2.6 at 673 K and *ZT*_average_ to 1.6 (323–723 K). Although the recent work reports a higher peak *ZT* (2.7) and *ZT*_average_ (1.7) via the strategy of high entropy in GeTe systems^40^, it contains 12% toxic Pb in the cation position, which makes it not comparable with this work in terms of environmentally friendly power-generation applications. Thus the reported thermoelectric performance in this work is competitive for lead-free GeTe-based polycrystal thermoelectrics.

## Results

### The atomic structure, electrical dipoles and potential distribution at QG position

**Fig. 2:**
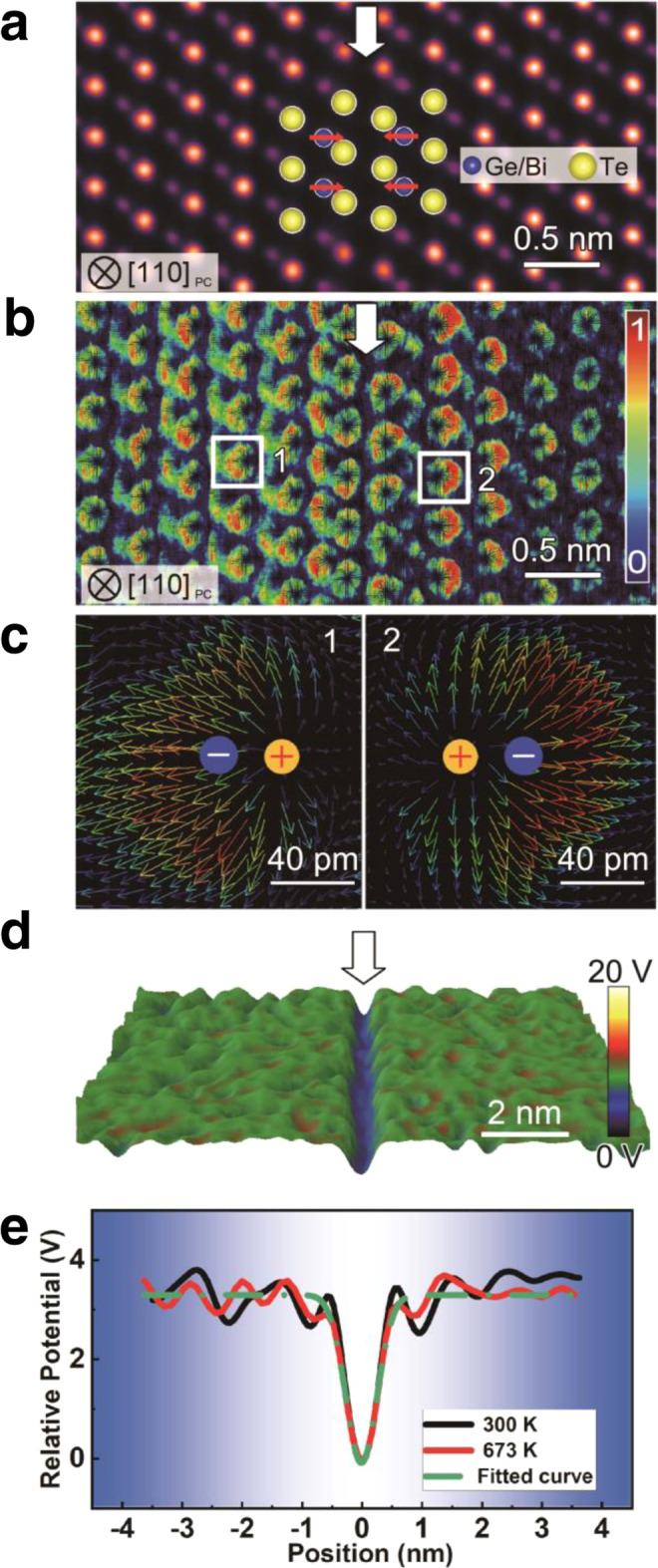
The structure of the QG. **a** An atomic-resolution HAADF image of a quantum gap in Ge_0.927_Bi_0.049_Te viewed along [110]_PC_ zone axis. **b** Local electric field of a quantum gap in Ge_0.927_Bi_0.049_Te deduced by differential phase-contrast imaging. **c** The magnified local electric fields from the atomic columns 1 and 2 in (**b**). **d** The reconstructed two-dimensional map of the potential distribution of a quantum gap in Ge_0.867_Re_0.003_Bi_0.087_Te by electron holography at 673 K. **e** The potential profiles across the potential well at 300 K and 673 K.

To understand the working mechanism of the QG, we first examine the structural base of QG in Ge_0.927_Bi_0.049_Te (Fig. 2). Fig. 2a displays an atomic-resolution image viewed along [110]_PC_, which clearly shows one missing layer of Ge atoms at the QG (white arrow). It is easy to find that Ge atoms near the QG move towards the QG, as demonstrated by the atomic models and red arrows. Besides, the Bi dopants are not ordered near QGs (see supplementary discussion and Supplementary Fig. 1 for details). To confirm the stability of the QG structure, in situ heating experiments were conducted from 300 to 723 K. The QGs (shown as linear grooves) do not change in the measured temperature range (Supplementary Fig. 2) indicating that they affect the thermoelectric properties from 300 to 723 K.

The state-of-the-art differential phase-contrast (DPC) imaging technique in STEM was applied to study the electric field (E-field) at the atomic scale^41–43^ (see Supplementary Fig. 3 for original data). The atoms near the QG have non-centrosymmetric E-fields (Fig. 2b). From the two enlarged E-field mapping results (boxes 1 and 2 in Fig. 2b), it can be seen that the positive and negative charge centers do not overlap (Fig. 2c), giving rise to electric dipoles^44^. These electric dipoles are along the direction away from the QG and their magnitudes gradually decay. To avoid the artifacts from scanning noise and sampling area, additional experiments were conducted (Supplementary Fig. 4) to confirm repeatability and that the results are consistent.

To corroborate the DPC results, in situ electron holography with varied temperatures under transmission electron microscopy (TEM) mode was also used to quantitatively investigate the potential of the QG^45,46^. The reconstructed potential of Ge_0.867_Re_0.003_Bi_0.087_Te at 673 K as a typical smoothed surface plot is shown in Fig. 2d, in which the potential well is indicated by the white arrow (original data for 300 K and 673 K can be seen in Supplementary Fig. 5 and Supplementary Fig. 6). The line profiles across the same QG at 300 K and 673 K prove that the potential at the QG area remains the same at elevated temperatures (Fig. 2e). The potential well was also verified by repeat experiments of Ge_0.927_Bi_0.049_Te (Supplementary Fig. 7), which confirms that the QGs in different samples are the same. The results of DPC and electron holography mutually confirm each other, which is further discussed in the supplementary discussion and depicted in Supplementary Fig. 8.

### The role of QG in electrical conductivity

**Fig. 3:**
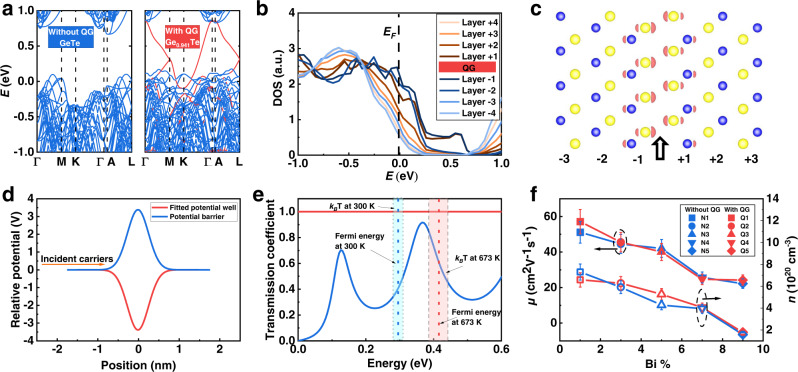
Theoretical analysis and experimental verification of the role of QG in electrical conductivity. **a** The band structure of GeTe with and without QG. The red color indicates the band states near QG. **b** Layer-resolved DOS of GeTe with QG. **c** The partial charge density around QG within the energy range of *E*_F_ ± 5*k*_B_*T* (*T* = 300 K). **d** The fitted quantum well and the corresponding quantum barrier. **e** The calculated transmission coefficients of the quantum well and quantum barrier. **f** The room-temperature mobilities and carrier concentrations of samples with and without QG with different Bi contents.

In such an atomic thin potential well, extra quantum states will be generated and they are numerically obtained by inputting the potential profile into a one-dimensional time-independent Schrödinger equation solver^47^. The energy states are already shown in Fig. 1b. The existence of quantum states is also confirmed by the density functional theory (DFT). The structural models for the DFT calculations are listed in Supplementary Fig. 9a, b. The band structure of GeTe with QG (Ge_0.941_Te) contains additional energy bands in contrast to that without QG (GeTe) (see red bands in Fig. 3a), which can also be seen in the total density of states (DOS) (Supplementary Fig. 9c). These additional energy bands are analogs to the quantum states in a single QG (Fig. 1b), which can be proved by the layer-resolved DOS near the QG. The adjacent Te and Ge atoms layers are counted as a unit and the calculated results show that the Ge-Te layers near QG have higher DOS near the Fermi level (*E*_*F*_) (Fig. 3b). The extra DOS near QG in real space can be seen more clearly in the energy-integrated partial charge (summed over range ±5 *k*_B_*T*, *T* = 300 K) (Fig. 3c).

The measured potential from electron holography in this work can be used to calculate the transmission coefficiency of charge carriers. In principle, the scattering process of charge carriers is controlled by the combination of Coulombic potential and exchange-correlation potential^48^. The former can be measured by electron holography under high accelerating voltage (>50 keV, 300 keV is applied in this work)^49^, while the latter can be calculated by the method in ref. 50. We find that the Coulombic potential is the absolute dominant potential since its magnitude is 19 orders of magnitude larger than the exchange-correlation potential (see supplementary discussion and Supplementary Fig. 10 for detail).

The Gaussian-shape quantum well together with the scattering states above the potential well result in the perfect transmission of carriers. We calculate the transmission coefficients (*T*_C_) of the experimentally obtained quantum well and a corresponding potential barrier for comparison (Fig. 3d). The *T*_C_-incident energy relationships in Fig. 3e show that the quantum well surprisingly allows perfect transmission, while the quantum barrier has a much lower and variable *T*_C_. The temperature affects the transmission coefficient by influencing the position of Fermi energy (*E*_F_, dependent on the carrier concentration) and the magnitude of thermal excitation *k*_B_T. But for QGs, the transmission coefficient is always near 1 (100%) from 300 K to 723 K (Fig. 3e). It should be noted that the Fermi energy here is roughly estimated by free carriers model for metals. The effective mass in the real case and the model to calculate the *E*_F_ will affect the position of *E*_F_. But from the *T*_C_-incident energy relationship, the QG always allows a perfect transmission only if Fermi energy is great than 0 (carrier concentration > 0), which is always satisfied. To check the reliability of our program, we calculate the *T*_C_ of a rectangle potential well using our program and the standard analytical result (only a simple quantum well has an analytical solution such as a rectangle well)^51^. The results of the two methods match very well (Supplementary Fig. 11).

The total relaxation time *t*_total_ can be determined by ${t}_{{{{{{\rm{total}}}}}}}^{-1}={t}_{{{{{{\rm{lattice}}}}}}}^{-1}+{t}_{{{{{{\rm{Bi}}}}}}}^{-1}+{t}_{{{{{{\rm{QG}}}}}}}^{-1}$^44^, where *t*_lattice_ is the relaxation time due to electron-phonon scattering; *t*_Bi_ is the relaxation time of Bi doping scattering; *t*_QG_ is the relaxation time of QG scattering. Since single QG allows near-perfect transmission, the mean free path (*λ*_QG_) between two effective scattering events by QGs will be extremely long, thus the corresponding relaxation time *t*_QG_ = *λ*_QG_/*v* (*v* is the drift velocity of carriers) is extremely large. In another word, the ${t}_{{{{{{\rm{QG}}}}}}}^{-1}$ term is extremely small that does not affect *t*_total_. Given the total carrier’s mobility is *μ*_total_ = e*t_total_/m^*^, where *e* is the carrier’s charge; m^*^ is the effective mass of carriers, it is therefore concluded that *μ*_total_ is marginally affected by QGs.

To verify our calculated results, we experimentally designed two groups of samples, which are labeled Q1–Q5 and N1–N5, separately. Under this design, the samples with the same number will have the same Bi content, but the Q group samples will contain QGs. We control the amount of QGs by controlling the number of Ge vacancies. The details of the composition and phase are presented in Supplementary Table 1 and Supplementary Fig. 12. The room-temperature mobilities (*μ*) are almost the same at each Bi content for the samples with and without QG (Fig. 3f and Supplementary Table 2). The high-temperature mobilities of two selected samples Q5 and N5 are the same as well (Supplementary Fig. 13). It can also be found that with the same Bi content, the DOS effective mass is not affected upon introducing the QG, as shown by the Pisarenko relationship of these ten samples (Supplementary Fig. 14).

### The role of QGs in lattice thermal conductivity

**Fig. 4:**
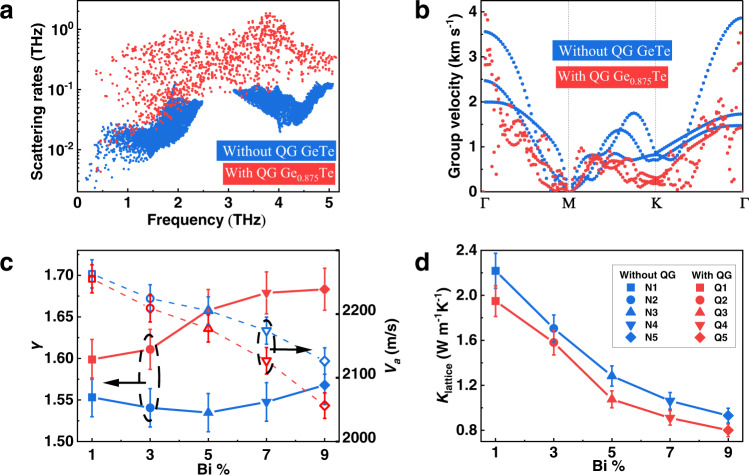
Theoretical analysis and experimental verification of the role of QGs in lattice thermal conductivity. **a** The calculated phonon scattering rates. **b** The calculated phonon group velocity. **c** The Grüneisen constant and average sound velocity of samples with and without QG. **d** Room temperature *κ*_lattice_ of samples with/without QG as a function of Bi content.

In the aspect of thermal transport, we expect the QGs dramatically lower the *κ*_lattice_ due to three different effects. First of all, the broken local translational symmetry and the van der Waals-like bonding nature of QG will enhance the bond anharmonicity at QG, leading to a higher anharmonic scattering rate^6^. In addition, the displacement of Ge atoms adjacent to the QG is increased, resulting in further loss of translational symmetries of the structures (Fig. 2a). Besides, the dipoles near QGs could contribute to anharmonic phonon scattering as well^52^. Secondly, the distribution of QGs in GeTe is not fully periodical. In other words, the non-periodicity of the QGs leads to an extremely large and complex cell that can also scatter phonons very effectively^53^. Based on anharmonic phonon calculations (see Supplementary Fig. 15 for the structural models and phonon spectrums), we indeed found that the anharmonic scattering rate of a large unit cell of GeTe with weak-bonded QG (Ge_0.875_Te) is stronger than that of GeTe without QG (GeTe) (Fig. 4a). It should be noted that QG density in the model for an anharmonic scattering rate calculation is higher than that under real conditions, which is a compromise due to the limited calculation resource. The last effect to lower lattice thermal conductivity is to modify the original phonon dispersion and lower the average phonon group velocity, as is shown in the calculated phonon dispersion results (Fig. 4b).

The promoted anharmonicity and lowered average phonon group velocity are consistent with experiments if we compare the Grüneisen constants (*γ*)^54^ and the average sound velocities between samples with and without QG (Fig. 4c and Supplementary Table 3). The samples with QG have higher Grüneisen constant (*γ*) and lower average sound velocity (*V*_*a*_). The measured *κ*_lattice_ of Q and N groups are also consistent with our conclusion (Fig. 4d). The samples with QG have lower *κ*_lattice_ than the samples without QG at each Bi content, as we expected.

### Improving final thermoelectric performance by introducing QG and adjusting their distribution

**Fig. 5:**
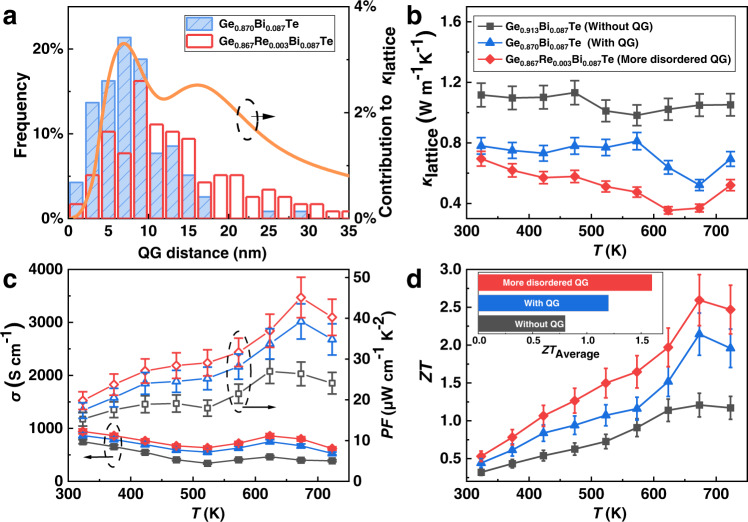
Improving the thermoelectric performance by modulating the distribution of QGs. **a** The histogram of QGs’ spacings and the corresponding mean-free-path resolved contribution to lattice thermal conductivity. **b** The lattice thermal conductivity. **c** The electrical conductivity and the Seebeck coefficient. **d** The *ZT* values and average *ZT* values.

An optimized *ZT* should consider both the QG and the Bi content. Since the QG is beneficial to *ZT*, it is suggested that the more the QGs in GeTe the better the thermoelectric performance. However, the fact is that the QG content cannot be too high for an optimized *ZT*. The reasons are as follows. The QGs are introduced by non-stoichiometric doping using materials such as Bi_2_Te_3_. However, the additional Bi atoms scatter the carriers and decrease the mobility. What’s more, the addition of Bi_2_Te_3_ decreases the carrier concentration which may deviate from the optimized value. Hence, the addition of Bi_2_Te_3_ should be controlled within a reasonable range for a lowered lattice thermal conductivity without introducing too many side effects. Here, we select an example of GeTe with QG (Ge_0.870_Bi_0.087_Te) to compare its structure and thermoelectric properties with the corresponding GeTe without QG (Ge_0.913_Bi_0.087_Te). Assuming all Ge vacancies are merged into QGs, Ge_0.870_Bi_0.087_Te contains 8.70% Bi and 4.35% QG (Supplementary Table 4). The structural information of Ge_0.870_Bi_0.087_Te and Ge_0.913_Bi_0.087_Te can be found in Supplementary Fig. 16a–c. From the measured thermoelectric properties shown in Fig. 5 and Supplementary Fig. 17, we find that both *σ* and *PF* increase while the *κ*_lattice_ decreases in Ge_0.870_Bi_0.087_Te (Fig. 5b, c). This is because of the increased carrier concentration (1.95 × 10^20^ cm^−3^ to 2.18 × 10^20^ cm^−3^) along with the introduction of QGs.

A broadened distribution of QGs’ spacings can further lower *κ*_lattice_.^55,56^ This can be achieved by the addition of the Re element (see supplementary discussion for details). For example, in Ge_0.867_Re_0.003_Bi_0.087_Te, the modulated distribution of QGs results in the lowered *κ*_lattice_ (Fig. 5a, b). The QGs in Ge_0.867_Re_0.003_Bi_0.087_Te are more disordered, as is indicated by the much diffuse distribution of the QGs’ spacings compared to Ge_0.870_Bi_0.087_Te (Fig. 5a) (see Supplementary Fig. 16d for the structural evidence). The disordered QG in Ge_0.867_Re_0.003_Bi_0.087_Te can increase the translational asymmetry, and modify the phonon spectrum, evidenced by the increased γ (from 1.68 to 1.75) and the lowered average sound speed compared with non-disordered QG in Ge_0.870_Bi_0.087_Te (from 2014 to 1980 m/s) (Supplementary Table 5). Besides, the more diffusely distributed QGs (Fig. 5a) can scatter phonons with a larger range of mean free paths (MFP) to further lower lattice thermal conductivity^55,56^, while not so diffusely distributed QGs can only scatter a part of these phonons. This mechanism is similar to applying all-scale defects to lower lattice thermal conductivity^57^. As a result, the *κ*_lattice_ of Ge_0.867_Re_0.003_Bi_0.087_Te is much lower than that of Ge_0.870_Bi_0.087_Te (Fig. 5b). Combining with Re’s effect on optimizing the carrier concentration to 6.86 × 10^20^ cm^−3^ and scattering phonons as point defects, the peak *ZT* improves to 2.6 and *ZT*_average_ improves to 1.6 (300–723 K) (Fig. 5d). The overall thermoelectric property is very competitive in lead-free polycrystal thermoelectrics. It is worth noting that the key factor to improve *ZT* comes from the disordered QG, other than the Re dopants. The GeTe with Re but without QGs (Ge_0.910_Re_0.003_Bi_0.087_Te) show much lower *ZT*, presumably due to the extremely low solubility of Re in GeTe lattice if the concentration of Ge vacancies is low. (Supplementary Fig. 19).

## Discussion

The decoupling of carrier and phonon transport from the QG can be attributed to the classical size effect and quantum size effect^6^. In this study, the quantum size effect is dominant. The classical size effect utilizes the mean-free-path difference of phonons and carriers to filter phonons. The phonons are distributed in a wide energy spectrum, thus they can be effectively scattered by all scale defects. While for carriers, only a narrow energy range near the Fermi level is involved in hole conduction such that the range of the mean free path is narrow. As a result, defects with proper size or separation can scatter phonons but partially allow carriers to pass through. The quantum size effect stems from the low-dimensional defects that generate quantum states near them and usually create a sharp DOS feature near *E*_*F*_ to increase the Seebeck coefficient^6^. But here, the atomic thin Gaussian-shape quantum well together with its scattering states result in the perfect transmission. We differentiate the classic and quantum size effects by comparing the QGs’ spacings to the MFP of holes. The MFP of the holes is about 10 nm for Ge_0.870_Bi_0.087_Te material at 10 K (see supplementary material for the calculation method), which is larger than most of the QGs’ spacings. This result again supports our conclusion that the QGs do not scatter the carriers. Thus, the quantum size effect must take effect.

We find the QGs can be extensively found in Ge deficient Ge–Bi–Te alloys after annealing. The roles of QGs in those QGs-containing Ge–Bi–Te alloys are similar as well. Two representative compounds are Ge_0.927_Bi_0.049_Te and Ge_0.867_Re_0.003_Bi_0.087_Te (Fig. 2, Supplementary Fig. 1–8). The former has fewer QGs inside (about 2.4%), while the latter has more QGs inside (about 4.4%). Although the density of QGs is different between Ge_0.927_Bi_0.049_Te and Ge_0.867_Re_0.003_Bi_0.087_Te, the measured potential profiles of QGs are similar. The above results indicates that the QGs can take effect in a series of Ge deficient Ge–Bi–Te alloys.

**Fig. 6:**
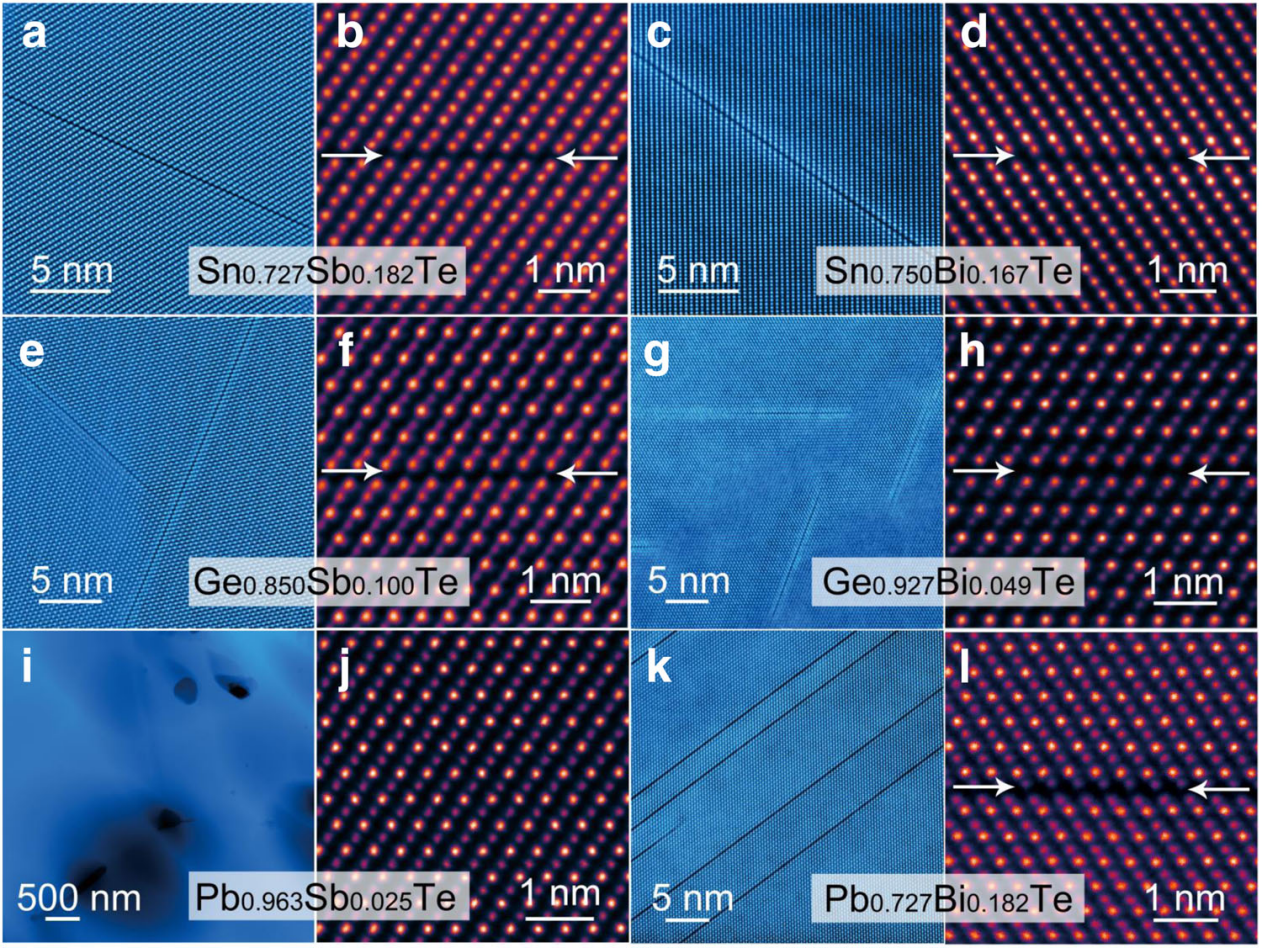
The library of QG-like structure in IVA-VIA compounds. The low magnification and high magnification images of Sn_0.727_Sb_0.182_Te (**a**, **b**) (*51*); Sn_0.750_Bi_0.167_Te (**c**, **d**); Ge_0.850_Sb_0.100_Te (**e**, **f**) (*28*); Ge_0.927_Bi_0.049_Te (**g**, **h**) *(29)*; Pb_0.963_Sb_0.025_Te (**i**) and (**j**); Pb_0.727_Bi_0.182_Te (**k**, **l**).

The introduction of QG by ordering vacancies is not limited to GeTe systems. Recent work reports that ordering vacancies can be introduced into SnTe by doping Sb_2_Te_3_ to boost thermoelectric performance^58^. The key to constructing QGs is to introduce enough vacancies by non-stoichiometric doping. With this understanding, we successfully introduce QG-like structure into SnTe, GeTe, and PbTe via doping Sb_2_Te_3_ and Bi_2_Te_3_. The experimental results are shown in Fig. 6. Apart from three reported materials^28,29,58^, we also successfully synthesized two new materials potentially containing QGs: Bi_2_Te_3_ doped SnTe and PbTe. These ordered cation vacancies are expected to work as QGs to decouple the carrier and phonon transport. As the local potential *V*_0_~1/Ω^45^, where Ω is the volume of the crystal unit cell, the ordered cation vacancies increase the Ω (the atom spacing increases) and thus decrease local potential, resulting in a potential well. This means that ordered vacancy layers are expected to generate QG.

Apart from ordered vacancies, any nanosized planar defects with nano-sized potential well may have a high transmission coefficient and the resultant decoupling effect. We compare the transmission coefficients of two different types of potential wells, i.e. Gaussian-shaped potential wells and wedge-shaped potential wells, with varied depth and width. As shown in Supplementary Figs. 20 and 21, the high transmission coefficients are not sensitive to the width and depth of the potential well. The above analysis shows that if there is a potential well by defects, it is highly likely to be QG. In contrast to potential wells, the transmission coefficients of potential barriers are much lower. The higher the barrier heights and the wider the widths, the lower the transmission coefficients^44^ (see the example of Gaussian-shaped potential barriers in Supplementary Fig. 22).

In summary, we found a class of defect ‘QGs’ can decouple the transport of carriers and phonons. The key feature of such QG is a nano-sized potential well around it which allows near-perfect transmission of carriers. Combining the effect of scattering phonons, the QG can realize a simultaneous decoupling of carriers and phonons transport. We find that the van der Waals gap in GeTe system is a representative example of such QG, since the atomic thin Gaussian-shaped quantum well formed at the van der Waals gap enables the near-perfect transmission of conducting carriers. By further disordering the distribution of QGs in GeTe, the *κ*_lattice_ can be lowered, leading to a peak *ZT* of 2.6 and a *ZT*_average_ of 1.6 (300–723 K). Finally, we also demonstrate that the concept of QG by introducing ordering vacancies can be extensively applied to other thermoelectric materials, such as the newly found QG-containing compounds Bi_2_Te_3_ doped SnTe and PbTe. We suggest that more general cases of QGs with nano-sized potential wells may be found beyond ordered vacancies and employed to decouple the carrier and heat transport.

## Methods

### Synthesis

The high-purity elements (Ge, Bi, Te) for a series of compositions were weighted and mixed into sealed quartz tubes. The quartz tubes were in a vacuum of about 10^−4^ Pa. The mixed materials were heated up to 673 K and kept for about two hours, then slowly heated to 1273 K for 12 h and hold for another 12 h, followed by water quenching. The afterword annealing process was at 873 K for two days. The ramp-up speed for each heating process was 1 K min^−1^. The produced ingots were ground into fine powders and sintered by Spark Plasma Sintering System (SPS-211LX) at 823 K under a pressure of 50 MPa for 5 min. The samples with and without QGs are treated with the same process as the above described.

### Physical Property Characterization

The electrical conductivity (*σ*) and Seebeck coefficient (*α*) were simultaneously measured by the Ulvac Riko ZEM-3 instrument with the uncertainty of 5% after cutting the samples into 2 × 2 × 12 cm^3^. The uncertainty of *PF* is thus 11%. The temperature-dependent Hall coefficients (*R*_H_) were measured by a Hall Effect measurement system (Lake Shore 8400 Series). The carrier concentration (n) is determined by 1/(*eR*_H_) and the carrier mobility (*µ*) is calculated as *σ*/(*en*). The uncertainty of n and *µ* is 10 and 11%, respectively. The room-temperature carrier concentration was cross-checked by the physical property measurement system (PPMS, Dynacool-14T, Quantum Design, U.S.A.) with a sweeping magnetic field range from −5 to 5 T.

We measure the thermoelectrical properties along the direction parallel to the press direction (in the process of spark plasma sintering) throughout this work. The thermal conductivity (*κ*) was calculated according to the equation: *κ* = *DC*_p_*d*. Here, D is the thermal diffusion coefficient and was measured by a Netzsch LFA467 equipment from 300 to 723 K with an uncertainty of 7%. Room temperature C_*p*_ of G and N group samples are measured by PPMS. High-temperature *C*_*p*_ is the specific heat capacity measured by STA 449 F3, Netzsch. The measured *C*_*p*_ values are offered in Supplementary Fig. 18. *d* is the density of each sample measured by Archimedes’ method. The uncertainty of final *ZT* is estimated to be 13%.

The sound velocities were measured at 298 K by the ultrasonic pulse-echo method using an Olympus 5073PR pulser/receiver. The echoes were received by a 5 MHz transducer and displayed on a Tektronix MDO3054 digital oscilloscope. The uncertainty of sound velocities is estimated as 1%.

### X-ray diffraction (XRD)

The phase purity and crystal structure were examined by the bulk X-ray diffraction with Cu-K_α_ radiation at room temperature. The machine is Smartlab (9 kW, Rigaku) diffractometer equipment.

### STEM/TEM

TEM specimens were prepared firstly by mechanical polishing down to the thickness of about 40 µm and then ion milled at the temperature of liquid nitrogen. The milling processes were at the beam voltages for 4.2 kV, 0.5 kV, and 0.1 kV, consequently with milling angles of ±5°. STEM measurements were performed on a Thermo Fisher Themis G2 60–300 electron microscope with an accelerating voltage of 300 kV. The convergence angle for the scanning beam was 25 mrad. The collection angles for the HAADF-STEM imaging were 70–200 mrad. To increase the signal-to-noise ratio, atomic-resolution HAADF-STEM images and Energy Dispersive Spectroscopy (EDS) mapping were obtained by averaging a consecutive series of drift-corrected images. DPC images were acquired by using segmented dark-field detectors. Before the DPC imaging experiment, the signal from four sectors of the detector has been adjusted to ensure linear responses to the beam current. The off-axis electron holography was carried out in TEM mode. The sample for electron holography was tilted away from the [110]_PC_ to more than 10 degrees to minimize the dynamic scattering effect. The phase and amplitude reconstruction and calculation of the mean inner potential were conducted using commercial software *HoloWorks v5.0*.

### Computational details

The calculations of electronic band structures are performed within density functional theory^59,60^, as implemented in the Vienna Ab-initio Simulation Package (VASP)^61^. Perdew–Burke–Ernzerhof (PBE) functional is considered in structural optimization^62^. The cut-off energy is 430 eV, and the corresponding k-grid is 14 × 14 × 2. The band structures are calculated by using a supercell of GeTe containing more than 110 atoms. The band decomposed partial charge densities are calculated in the energy range of *E*_F_ ± 0.13 eV for Fig. 3c. The scattering calculations of carriers by quantum well refer to the scattering problem section of *introduction to quantum mechanics*^51^, more details can be seen in supporting materials. The calculations of thermal transport properties are based on the temperature-dependent effective potential (TDEP) method. Our supercell for thermal property calculation of GeTe with QG contains 14% QG, which means one of eight Ge atomic layers is removed. For QG-GeTe and pure GeTe, 240 atoms and 234 atoms supercells are used in molecular dynamics (MD) simulation. More details are given in supplementary materials. The scattering rates of QG-GeTe are calculated with 11 × 11 × 2 q-point mesh for Brillouin zone integrations. To verify the accuracy of our results, we also compare our results for pure GeTe with literature^63^. Limited by the current computing resources, we choose the nearest neighbor radius as large as possible to calculate the force constants. The cut-offs for the 2nd and 3rd interatomic force constants are 8.3 Å and 7.0 Å (up to the 15th nearest neighbors) for QG-GeTe and 7.8 Å and 6.8 Å for pure GeTe, respectively. The phonon dispersions of QG-GeTe and pure GeTe, which include all the acoustics and optics branches, are provided in the supplementary materials.

## Supplementary information

Supplementary Information

## Data Availability

The authors declare that the data supporting the findings of this study are available within the paper and its Supplementary Information file. All of the other data are available from the authors upon reasonable request.
