# Supplementary material for: Tunable quantum gaps to decouple carrier and phonon transport leading to high-performance thermoelectrics
Source: Nat Commun. 2022 Sep 24;13:5612. doi: 10.1038/s41467-022-33330-9 (PMC9509343; doi:10.1038/s41467-022-33330-9)
Supplement: Supplementary file 1 — Supplementary Information [file 41467_2022_33330_MOESM1_ESM.pdf]

# Tunable quantum gaps to decouple carrier and phonon transport leading to high-performance thermoelectrics

Yong Yu[1,2,5], Xiao Xu[1,5], Yan Wang[1,5], Baohai Jia[1], Shan Huang[1], Xiaobin Qiang[1], Bin Zhu[1], Peijian Lin[1], Binbin Jiang,[1] Shixuan Liu[1], Xia Qi[3], Kefan Pan[1], Di Wu[3], Haizhou Lu[1], Michel Bosman[2], Stephen J. Pennycook[2], Lin Xie [1]* and Jiaqing He[1,4]*

[1]Shenzhen Key Laboratory of Thermoelectric Materials, Department of Physics, Southern University of Science and Technology, Shenzhen, 518055, China.

[2]Department of Materials Science and Engineering, National University of Singapore, 117575, Singapore.

[3]School of Materials Science and Engineering, Shaanxi Normal University; Key Laboratory for Macromolecular Science of Shaanxi Province, Xi'an 710062, China

[4]Guangdong-Hong Kong-Macao Joint Laboratory for Photonic-Thermal-Electrical Energy Materials and Devices, Southern University of Science and Technology, Shenzhen 518055, China

[5] These authors contributed equally: Yong Yu, Xiao Xu, Yan Wang

# Table of Contents

Supplementary Discussion ........ 4

The relationship of the distribution of Bi with the QG ........ 4

Technical detail of DPC ........ 4

Technical detail of (*in-situ*) Electron Holography ........ 5

The relationship between the dipoles and potential well ........ 5

Scattering problem of arbitrary potential. ........ 6

Cross-check the carrier concentrations of Q5 and N5 samples ........ 7

The effect of QG on the Seebeck coefficient ........ 7

Theory basis for analyzing lattice thermal conductivity ........ 7

Sound velocity and Grüneisen parameter ........ 8

Calculation of lattice thermal conductivity ........ 8

The origin of the disordered QGs ........ 9

Estimation of hole mean-free-path ........ 9

Supplementary Figures ........ 11

Supplementary Fig. 1. The EDS map of Bi segregation near QGs ........ 11

Supplementary Fig. 2. The *in-situ* heating TEM images of $Ge_{0.867}Re_{0.003}Bi_{0.087}Te$ sample ........ 12

Supplementary Fig. 3. Raw DPC images of $Ge_{0.927}Bi_{0.049}Te$. ........ 13

Supplementary Fig. 4. Repeat experiments of the E-field mapping of $Ge_{0.927}Bi_{0.049}Te$ ........ 14

Supplementary Fig. 5. *In-situ* electron holography of a representative QG in $Ge_{0.867}Re_{0.003}Bi_{0.087}Te$. ........ 15

Supplementary Fig. 6. The quantum well of QG at 300 K and 673 K in $Ge_{0.867}Re_{0.003}Bi_{0.087}Te$ ........ 16

Supplementary Fig. 7. Repeat experiments of the potential mapping from 6 different areas a-f of $Ge_{0.927}Bi_{0.049}Te$ at room temperature ........ 17

Supplementary Fig. 8. The origin of macro-E-field in $Ge_{0.927}Bi_{0.049}Te$. ........ 18

Supplementary Fig. 9. The structural models for DFT calculations and electronic DOS of GeTe with QG ($Ge_{0.941}Te$) and without QG (GeTe). ........ 19

Supplementary Fig. 10. The $V$xc dependence on carrier concentration ........ 20

Supplementary Fig. 11. The transmission coefficient calculated by the numerical method in this work compared with the standard analytical method. ........ 21

Supplementary Fig. 12. X-ray diffraction (XRD) patterns of Q and N group samples. ........ 22

Supplementary Fig. 13. The temperature-dependent carrier concentration and mobility of Q5 and N5 samples ........ 23

Supplementary Fig. 14. The Pisarenko relationship of Q and N group samples. ........ 24

Supplementary Fig. 15. The structural models for molecular dynamics simulations and phonon dispersions of GeTe without QG (GeTe) and GeTe with QG ($Ge_{0.875}Te$) ........ 25

Supplementary Fig. 16. Structure characterizations of $Ge_{0.913}Bi_{0.087}Te$ (without QG), $Ge_{0.870}Bi_{0.087}Te$ (with QG), and $Ge_{0.867}Re_{0.003}Bi_{0.087}Te$ (more disordered QG) for comparison ........ 26

Supplementary Fig. 17. Seebeck coefficients and total thermal conductivity of $Ge_{0.913}Bi_{0.087}Te$ (without QG), $Ge_{0.870}Bi_{0.087}Te$ (with QG), and $Ge_{0.867}Re_{0.003}Bi_{0.087}Te$

(more disordered QG) ........................................................................................27
Supplementary Fig. 18. Measured Cp values of Ge0.$_{913}$$Bi_{0.087}$Te (without QG), $Ge_{0.870}Bi_{0.087}Te$ (with QG), and $Ge_{0.867}Re_{0.003}Bi_{0.087}Te$ (more disordered QG). ..............28
Supplementary Fig. 19. The effect of Re and QG on thermoelectric properties .............29
Supplementary Fig. 20. The Gaussian-shaped potential wells with varied depth and width and the corresponding transmission coefficients. ..........................................................30
Supplementary Fig. 21. The wedge-shaped potential wells with varied depth and width and the corresponding transmission coefficients ............................................................31
Supplementary Fig. 22. The Gaussian-shaped potential barriers with varied depth and width and the corresponding transmission coefficients ..................................................32
Supplementary Tables ...........................................................................................................33
Supplementary Table 1 The compositions of Q and N group samples ...........................33
Supplementary Table 2 Repeated carrier concentrations and mobilities .........................34
Supplementary Table 3 Sound velocities (longitudinal sound velocity $v_l$, transverse sound velocity $v_t$, average sound velocity $v_a$) of Q and N group samples. ...............................35
Supplementary Table 4 The compositions of samples for *ZT* enhancement. ..................36
Supplementary Table 5 The longitudinal sound velocity $v_l$, transverse sound velocity $v_t$, average sound velocity $v_a$, the poisson ratio r and the Grüneisen parameter of samples for *ZT* enhancement. ..........................................................................................................37

**Supplementary Discussion**

The distribution of Bi

The QGs stem from ordered Ge vacancies[1], which are mainly introduced by $Bi_2Te_3$ in this work:

$$Bi_2Te_3 \xrightarrow{3GeTe} 2Bi_{Ge}^{\cdot} + V_{Ge}'' + 3Te_{Te}^{x} \qquad \text{(eq. S1)}$$

The Bi elements may segregate near some QGs due to defect reaction, which will show a brighter contrast in the HAADF image (the QG labeled as N in Supplementary Fig. 1a). But not all the QGs (the QG labeled as M) have this kind of brighter contrast (Supplementary Fig. 1a.) The missing layer of Ge causes the darker contrast of Ge mapping in Supplementary Fig. 1b. The closer Te layer due to the missing of Ge layer causes the brighter contrast of Te mapping in Supplementary Fig. 1c. The Bi content is 1.5% higher at the QG-N and it gives rise to the brighter contrast of the HAADF image. But QG-M does not have higher Bi content (Supplementary Fig. 1d and e). The EDS spectrum (Supplementary Fig. 1f) proves that all the above mappings are based on signals from strong peaks.

The Bi elements near QGs may be mildly segregated but not fully ordered. The amount of Bi doping and the vacancies should have a rough relationship of 2:1 according to eq. S1. If the Bi atoms are ordered near QGs, one layer of QG will cause the ordering of two-layer Bi (two sides of QGs), which will result in a sharp drop to almost zero of Bi content away from the QG, but the line scan across the QG indicates that Bi elements are almost constant away from the QG (Supplementary Fig. 1e).

In summary, the Bi may have mild segregation near some QGs but not completely ordered.

Technical detail of DPC

Differential Phase Contrast (DPC) imaging is used in STEM to directly measure local electromagnetic fields in materials. The converged electron beam deflected by the electric field will generate a differential signal which is recorded by the segmented detector. By analyzing the differential signal, we can map the electric field distribution.

The raw data in this work are shown in Supplementary Fig. 3.

To exclude the effect of scanning noise, we apply DPC to the same area from two orthogonal directions (Supplementary Fig. 4a and b). We have also mapped six random areas (Supplementary Fig. 4c-h), and the same result is obtained.

Technical detail of (*in-situ*) Electron Holography

The original data for potential mapping of $Ge_{0.867}Re_{0.003}Bi_{0.087}Te$ are shown in Supplementary Fig. 5. We use the hologram (Supplementary Fig. 5a), and reference (Supplementary Fig. 5b) images to obtain the amplitude (Supplementary Fig. 5c) and phase (Supplementary Fig. 5d). Then we can calculate the mean inner potential (Supplementary Fig. 6a). Same procedures are conducted to Supplementary Fig. 5e-h to obtain the potential mapping (Supplementary Fig. 6b). The obtained potential profiles are shifted together by aligning the lowest point of potential to zero.

Six potential mappings are obtained in $Ge_{0.927}Bi_{0.049}Te$ (Supplementary Fig. 7) using the same methods. The highly repeatable data prove that the results of quantum wells at QG are reliable. We obtain the same results of quantum well in two QG containing GeTe materials. This means that the QGs in different Ge-Bi-Te compounds have the same potentials.

The relationship between the dipoles and potential well

The electric dipoles as observed by DPC imaging are caused by macroscopic E-field ($E_{macro}$), which is the gradient of the potential well (Supplementary Fig. 8). In GeTe lattice without QG, the positively charged Ge layer and negatively charged Te layer stack alternatively, such that the E-field from Ge layer to Te layer is canceled by the E-field from Te layer to Ge layer (Supplementary Fig. 8a). In this way, there is no net E-field on the macroscopic scale, which lowers the total electrostatic potential energy. However, the QG breaks the balance and net macro-E-field appears (Supplementary Fig. 8b). The directions of $E_{macro}$ are toward the QG. The Te and Ge atoms near the QG are strongly polarized by this $E_{macro}$. The polarized local E-field

($E_{local}$) can be calculated by equation[2]:

$$\boldsymbol{E_{local}} = -\frac{\mathbf{1}}{\mathbf{3}}(\boldsymbol{\varepsilon_r} + \mathbf{2}) * \boldsymbol{E_{macro}}, \qquad \text{(eq. S2)}$$

where $E_{local}$, $E_{macro}$ and $\varepsilon_r$ are the local E-field, macro-E-field, and relative dielectric constant. The $\varepsilon_r$ of GeTe is about 36 to 58[3,4]. As a result, $E_{\mathrm{local}}$ is at least 13 times stronger than the $E_{\mathrm{macro}}$. Thus, the observed E-field from the DPC is mainly from $E_{local}$ by dipoles (Supplementary Fig. 8c).

Calculation of exchange-correlation potential.

The exchange-correlation potential $V_{\mathrm{XC}}$ can be calculated by[5]

$$V_{\mathrm{xc}} = -0.985\frac{e^2}{4\pi\varepsilon_0}n^{1/3}\left\{1 + \frac{0.0334}{a_{\mathrm{B}}^{*}n^{1/3}}\ln\left[1 + 18.376a_{\mathrm{B}}^{*}n^{1/3}\right]\right\}^{5} \text{(eq. S3)}$$

where $a_{\mathrm{B}}^{*}$ is the effective Bohr radius for effective electron mass, $\varepsilon_0$ is the dielectric constant, and $n$ is the carrier concentration. We plot the calculated $V_{\mathrm{xc}}$ dependence on carrier concentration in and find the magnitude of $V_{\mathrm{xc}}$ is at the level of $10^{-19}$ V which is far lower than the magnitude of Coulombic potential at the level of 8V (Fig. 2e).

Scattering problem of arbitrary potential.

The conduction electrons in the semiconductor can be well described by following Hamiltonian

$$H = \frac{\hbar^2}{2m}k^2 + U, \qquad \text{(eq. S4)}$$

where $\hbar$ is the reduced Planck constant, $m$ is the effective mass, $k$ is the wave-vector, and $U$ is the scattering potential. For arbitrary $U$, it is impossible to analytically solve this problem and numerical method is used. Firstly, by using the discrete Fourier transformation we get the tight-binding Hamiltonian in the real space,

$$H = t\sum_i c_i^{\dagger}c_{i+1} - 2t\sum_i c_i^{\dagger}c_i + \sum_i U_i c_i^{\dagger}c_i \qquad \text{(eq. S5)}$$

where $t = \hbar^2/2ma^2$, $a$ is the distance of the discrete lattice, and $c^{\dagger}$ and $c$ are creation and annihilation operators. To obtain the transmission coefficient $T$, we use the Landauer-Büttiker formula[6] and the recursive Green function method[7] , which are frequently used in transport problem[8–10].

$$T = \mathrm{Tr}\,[\Gamma_l G^R \Gamma_r G^A] \qquad \text{(eq. S6)}$$

where $G^{R/A} = 1/(E - H - \Sigma_l^{R/A} - \Sigma_l^{R/A}))$ is the retarded/advanced Green function, $E$ is the incident energy. $\Gamma_{l/r} = i(\Sigma_{l/r}^R - \Sigma_{l/r}^A)$ is the linewidth function and $\Sigma_{l/r}^{R/A}$ is the retarded/advanced self-energy of the left/right free region, which can be defined as

$$\Sigma_{l/r}^{R/A} = t^2 g_{l/r}^{R/A} \qquad \text{(eq. S7)}$$

where $g_{l/r}^{R/A} = 1/(E - H_0 \pm i\eta)$ is the surface Green function of semi-infinite free region, $H_0 = t\sum_i c_i^\dagger c_{i+1} - 2t\sum_i c_i^\dagger c_i$ is the free Hamiltonian, $\eta$ is an infinitesimal number.

Cross-check the carrier concentrations of Q5 and N5 samples.

The accuracy of carrier mobility depends on the accuracy of electrical conductivity and carrier concentration, in which the carrier concentration is sensitive to the experimental condition. Here we cross-check the carrier concentration of Q5 and N5 samples by PPMS, and the results of carrier concentrations are $1.63 \times 10^{20}$ $cm^{-3}$ and $1.49 \times 10^{20}$ $cm^{-3}$, respectively. The calculated mobilities are 26.56 $cm^2V^{-1}s^{-1}$ and 23.5 $cm^2V^{-1}s^{-1}$, respectively.

The effect of QG on the Seebeck coefficient

From the calculated DOS of GeTe (without QG) and $Ge_{0.941}Te$ (with QG), we find the QG does not introduce strong DOS distortion near Fermi level, which should lead to almost unchanged DOS effective mass (Supplementary Fig. 9). The above analysis is consistent with the experimental results. After the addition of QG, the DOS effective mass keeps almost constant (Supplementary Fig. 13). Therefore, the lower Seebeck coefficients seen in Q group samples are mainly due to the higher carrier concentration than N group samples.

Calculation of lattice thermal conductivity

According to phonon Boltzmann transport theory, the lattice thermal conductivity

can be expressed as[11]

$$\kappa_{lattice} = \frac{1}{N\Omega k_B T^2}\sum_q \frac{n_q(n_q+1)\omega_q^2 v_q{}^2}{s_q}, \qquad \text{(eq. S8)}$$

where $N$, $\Omega$, $n_q$, $\omega_q$, $s_q$, and $v_q$ are the total number of phonon states $q$, volume, phonon distribution function, angular frequency, scattering rates, and group velocity, respectively. The dominant parameters are $s_q$ and $v_q$. For a low $\kappa_{lattice}$, the desired $s_q$ should be large while the $v_q$ should be small.

Sound velocity and Grüneisen parameter

The average sound velocity ($v_a$) is calculated from the sound velocity as[12].

$$\frac{1}{v_a} = \left[\frac{1}{3}\left(\frac{1}{v_l^3} + \frac{2}{v_t^3}\right)\right]^{1/3}, \qquad \text{(eq. S9)}$$

where $v_l$ is the longitudinal sound velocity and $v_t$ is the transverse sound velocity. Poisson ratio ($r$) is calculated by[13]

$$r = \frac{1-2(v_t/v_l)^2}{2-2(v_t/v_l)^2}. \qquad \text{(eq. S10)}$$

The Gruneisen parameter ($\gamma$) is calculated by[12]

$$\gamma = \frac{3}{2}\left(\frac{1+r}{2-3r}\right). \qquad \text{(eq. S11)}$$

Calculation of lattice thermal conductivity

The following formula is frequently used to calculate the Lorentz constant and the electron thermal conductivity[14–16].

$$L = 1.5 + \exp\left[-\frac{|S|}{116}\right], \qquad \text{(eq. S12)}$$

$$\kappa_{latt} = \kappa - \kappa_e = \kappa - L\sigma T. \qquad \text{(eq. S13)}$$

However, this is not accurate enough when the phonon scattering mechanism is not dominant. The accuracy can be improved according to the following formula.

The carrier concentration is

$$n = \int_0^\infty g(E) f_0(E)\,dE = \frac{N_v}{2\pi^2}\left(\frac{2m_d^*}{\hbar^2}\right)^{\frac{3}{2}} \int_0^\infty \frac{E^{\frac{1}{2}}}{e^{(E-E_F)/k_B T}+1} dE. \qquad \text{(eq. S14)}$$

For the sake of simplicity, the Fermi integrals are defined as

$$F_s = \int_0^\infty \frac{E^{*s}}{e^{(E^*-E_F^*)}+1} dE^*. \qquad \text{(eq. S15)}$$

The thermoelectric transport coefficients become:

$$n = \frac{N_v}{2\pi^2}\left(\frac{2m_d^* k_B T}{\hbar^2}\right)^{\frac{3}{2}} F_{1/2}, \quad \text{(eq. S16)}$$

$$\sigma = \frac{N_v e^2 \tau_0}{3\pi^2 m_c^*}\left(\frac{2m_d^* k_B T}{\hbar^2}\right)^{\frac{3}{2}}\left(r+\frac{3}{2}\right) F_{r+1/2}, \quad \text{(eq. S17)}$$

$$S = -\left(\frac{k_B}{e}\right)\left[\frac{\left(r+\frac{5}{2}\right)F_{r+3/2}}{\left(r+\frac{3}{2}\right)F_{r+1/2}} - E_F^*\right], \quad \text{(eq. S18)}$$

$$\boldsymbol{L_o} = \left(\frac{\boldsymbol{k_B}}{\boldsymbol{e}}\right)^2 \left\{\frac{\left(r+\frac{7}{2}\right)\boldsymbol{F}_{r+5/2}}{\left(r+\frac{3}{2}\right)\boldsymbol{F}_{r+1/2}} - \left[\frac{\left(r+\frac{5}{2}\right)\boldsymbol{F}_{r+3/2}}{\left(r+\frac{3}{2}\right)\boldsymbol{F}_{r+1/2}}\right]^2\right\}. \quad \text{(eq. S19)}$$

Scattering mechanisms are generally divided into three types: acoustic phonon scattering (APS) ($r = -\frac{1}{2}, \sigma \propto T^{-3/2}$), polar optical phonon scattering (OPS) ($r = \frac{1}{2}, \sigma \propto T^{-1/2}$), and ionized impurity scattering (IIS) ($r = \frac{3}{2}, \sigma \propto T^{3/2}$).

The three corresponding lattice thermal conductivity have different relations with temperature. It is found that APS plays a dominant role in GeTe for Q1, Q2, and Q3, and thus $r = -\frac{1}{2}$. However, when there are more impurities, the impurity scattering increases, and the conductivity curve is irregular. Therefore, for group 4 and group 5 samples, there are two kinds of scattering (APS and IIS). For Group 4, $r = \frac{3}{2}$ and $r = -\frac{1}{2}$ are used at low temperature and high temperature, respectively. For Group 5, $r = \frac{3}{2}$.

<u>The origin of the disordered QGs.</u>

For the $Ge_{0.870}Bi_{0.087}Te$, the QGs are formed by homogeneous nucleation, thus their spacings are random distributed with a peak at around 7 nm, while for Re-doped GeTe the Re-dopants produce a strong strain field due to the huge atomic radius difference between Re and Ge, (Re:188pm, Ge:125pm)[17], which facilitates the inhomogeneous nucleation of QG and affect its distribution. The result is that the distribution is broadened. The Bi dopants can not play such a strong role as inhomogeneous nucleation center because of its similar atomic radius (143 pm)[10].

<u>Estimation of hole mean-free-path.</u>

The hole velocity from DFT is $0.23\times 10^6$ m/s. The mobility and effective mass from experiments are 27 $cm^2V^{-1}s^{-1}$ at 10 K and 2.8 $m_0$, respectively. The relaxation time of hole is

$$\tau = \frac{\mu m_c^*}{e} = 43 \text{ fs}, \qquad \text{(eq. S20)}$$

and the hole mean-free-path is

$$\Lambda \approx v_F \tau = 10 \text{ nm}. \qquad \text{(eq. S21)}$$

## Supplementary Figures

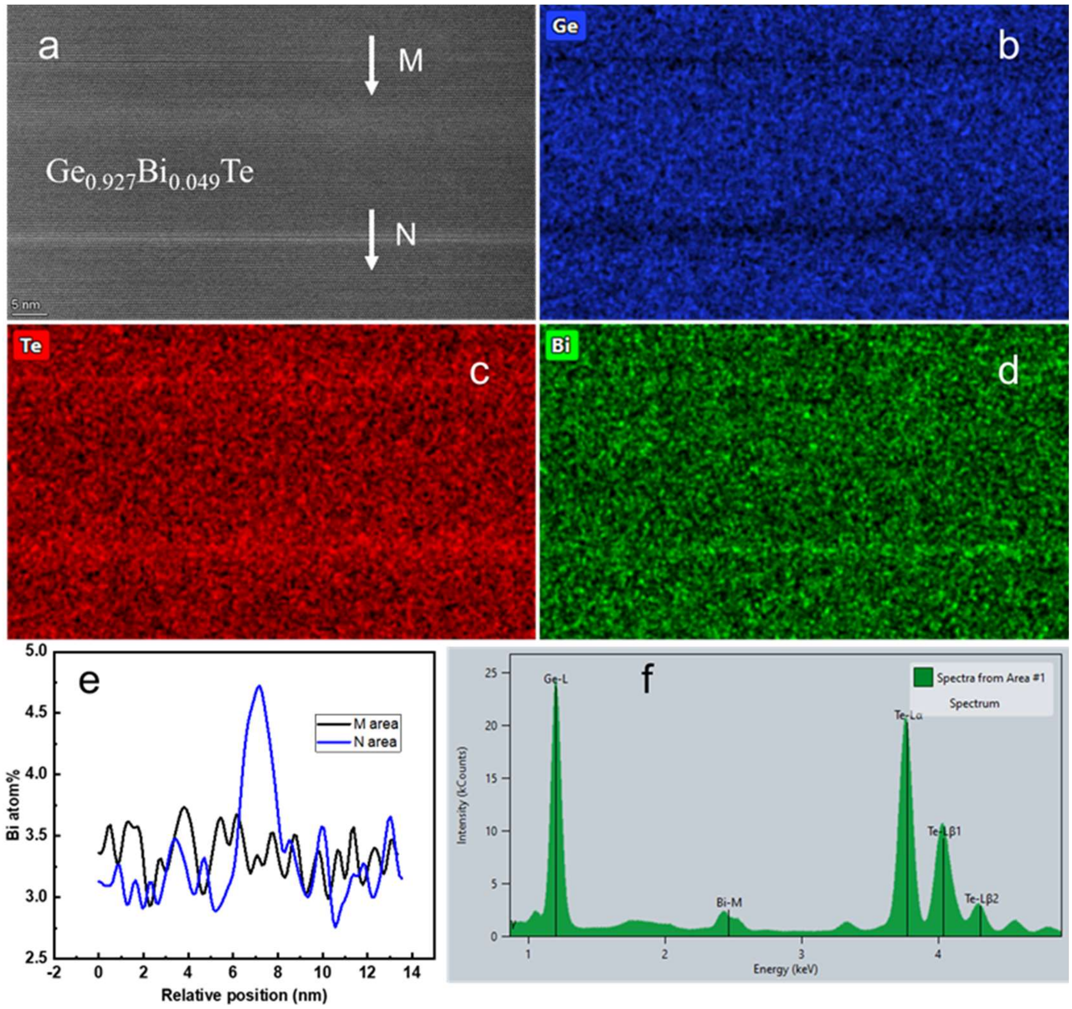


**Supplementary Fig. 1. The EDS map of Bi segregation near QGs. a**, the HAADF image of the studied area. **b, c, d** are the elemental mapping of Ge,Te and Bi elements. Brightness is set to proportional to the atomic ratio of each element**. e**, the averaged line profiles across the two QGs M and N, as indicated by the white arrows in **a**. **f**, the EDS spectrum of the studied area.

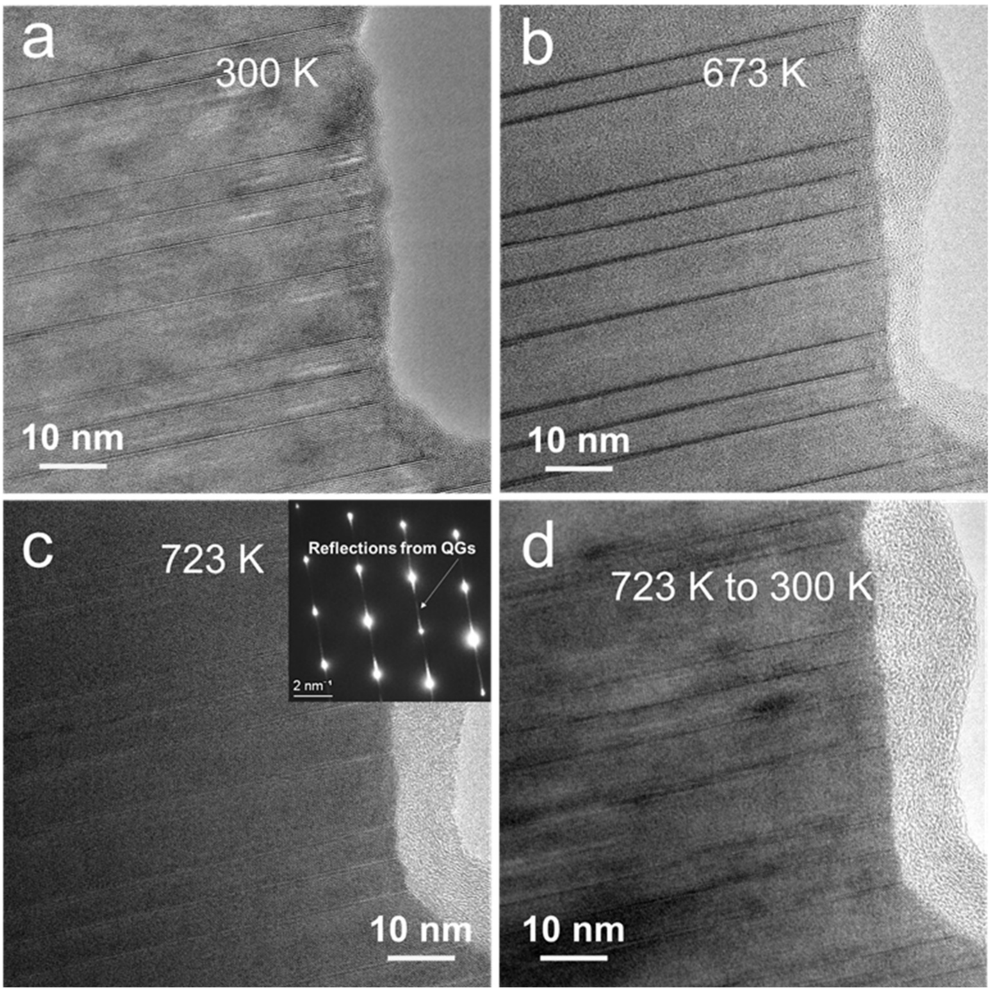


**Supplementary Fig. 2. The *in-situ* heating TEM images of $Ge_{0.867}Re_{0.003}Bi_{0.087}Te$ sample.** The temperatures are **a**, 300 K, **b**, 673 K, **c**, 723 K, and **d**, 723 K cooling to 300 K. The inset in **c** is the selected area electron diffraction at 723 K.

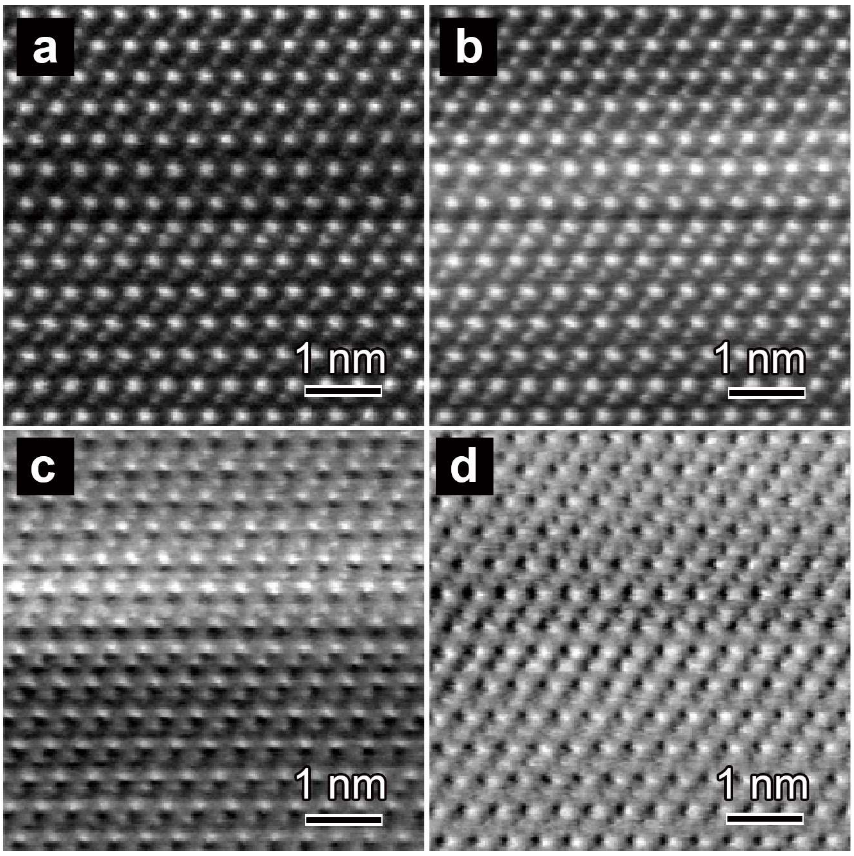


**Supplementary Fig. 3. Raw DPC images of $Ge_{0.927}Bi_{0.049}Te$. a**, The HAADF image (collection angle 120-200 mrad). **b**, The ADF image (collection angle: 29-113 mrad). **c**, DPCx Signal of A-C. **d**, DPCy signal of B-D.

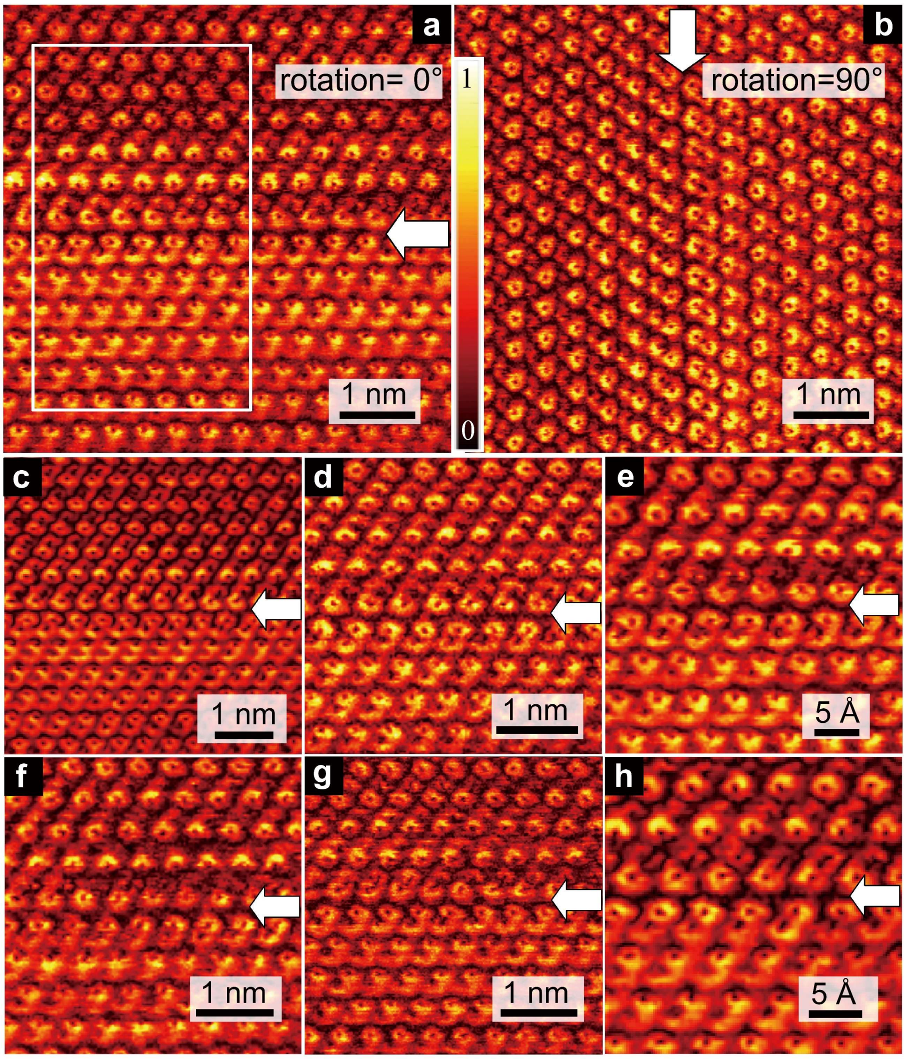


**Supplementary Fig. 4. Repeat experiments of the E-field mapping of $Ge_{0.927}Bi_{0.049}Te$. a**, **b** are from the same area but the scanning directions are perpendicular to each other. The white rectangle indicates the area shown in Fig. 2b. Inserted intensity bar shows the relative E-field strength. **c-h** are E-field mapping from six different areas. The position of QGs are indicated by white arrows.

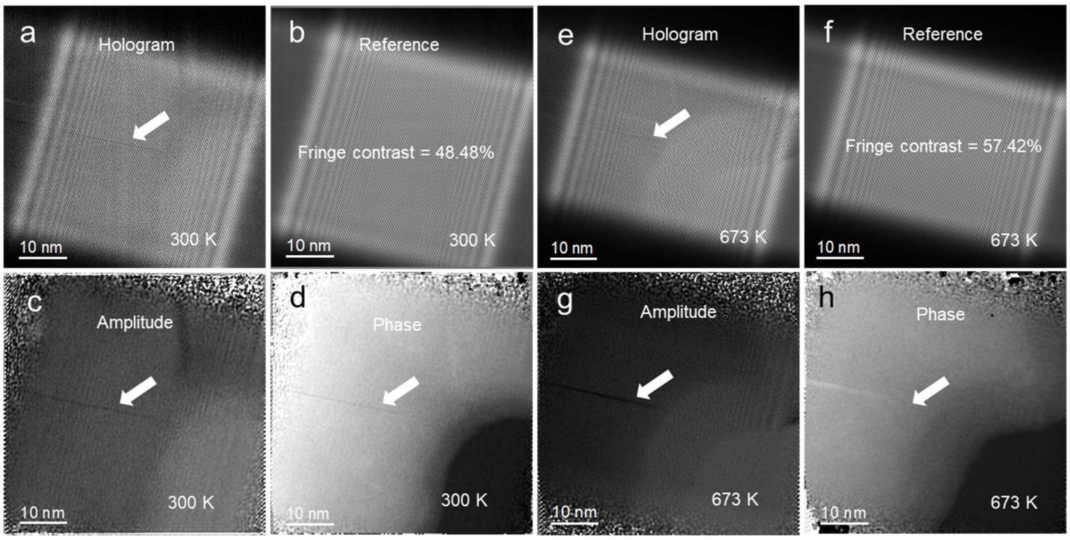


**Supplementary Fig. 5. *In-situ* electron holography of a representative QG in $Ge_{0.867}Re_{0.003}Bi_{0.087}Te$. a-d** are the data at 300 K: **a**, The hologram. **b**, The reference with a fringe contrast of 48.48%. **c**, The calculated amplitude. **d**, The calculated phase. **e-h** are the data acquired at 673 K: **e**, The hologram. **f**, The reference with a fringe contrast of 57.42%. **g**, The calculated amplitude. **h**, The calculated phase.

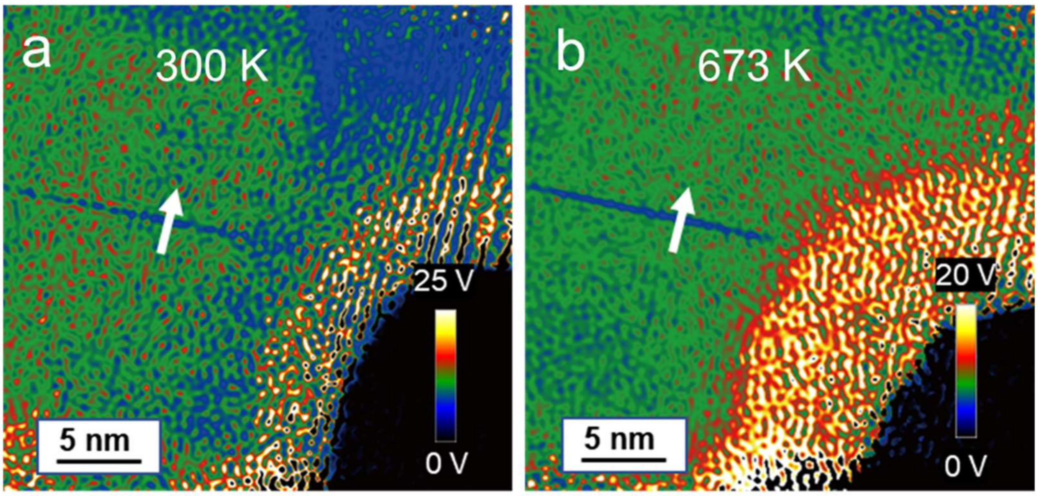


**Supplementary Fig. 6. The quantum well of QG at 300 K and 673 K in $Ge_{0.867}Re_{0.003}Bi_{0.087}Te$. a,** The potential mapping at 300 K. **b,** The potential mapping at 673 K.

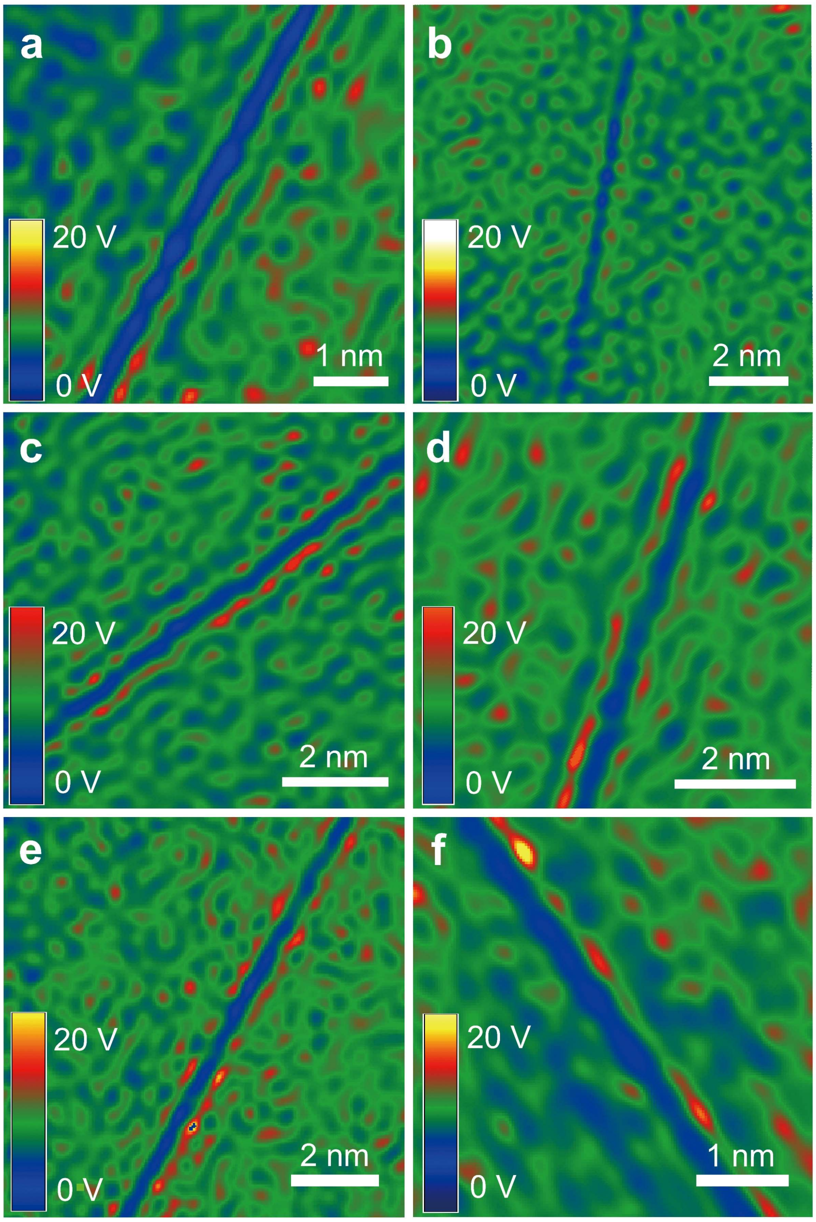


**Supplementary Fig. 7. Repeat experiments of the potential mapping from 6 different areas a-f of $Ge_{0.927}Bi_{0.049}Te$ at room temperature.**

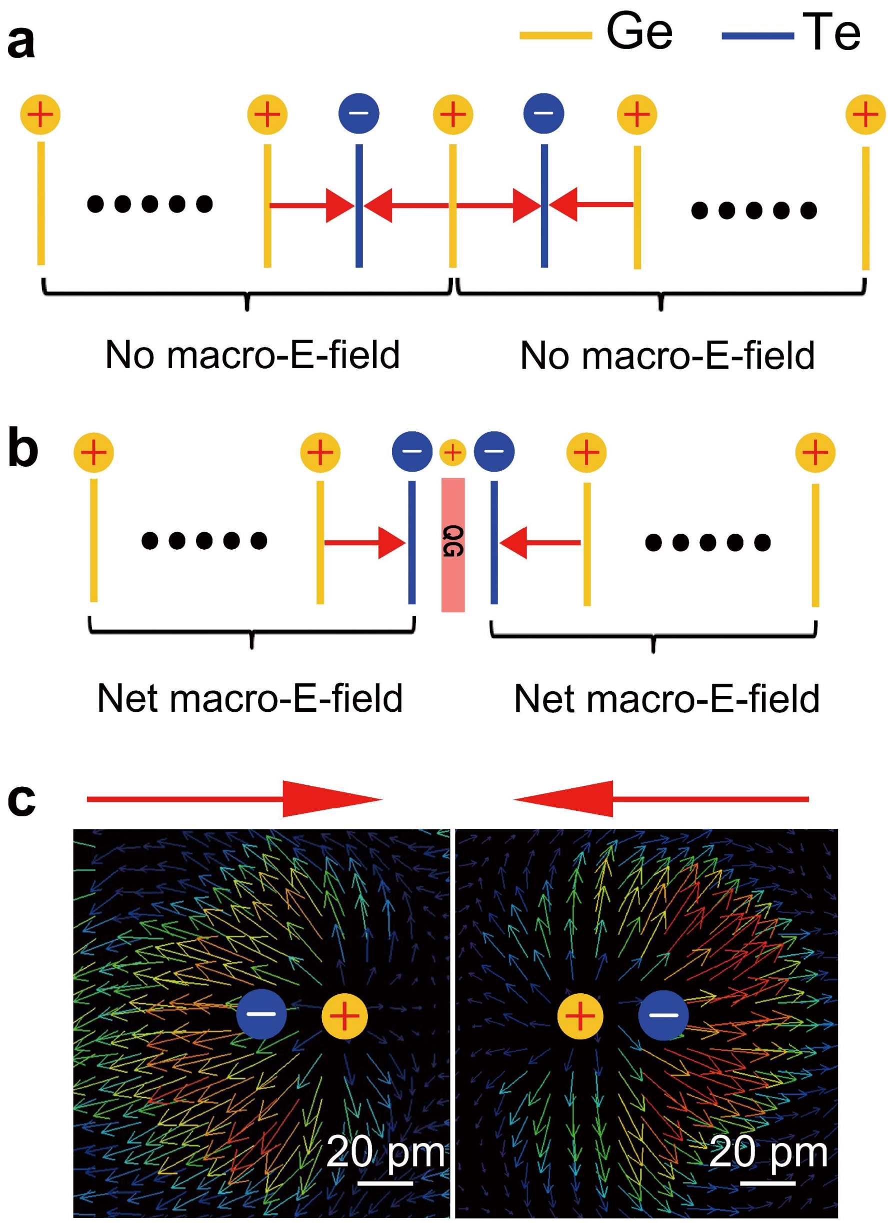


**Supplementary Fig. 8. The origin of macro-E-field in $Ge_{0.927}Bi_{0.049}Te$. a,** The balance E-field between positive charged Ge layer and negative charged Te layer. **b**, The net macro-E-field due to the missing of the positively charged Ge layer. **c**, The relationship between the macro-E-field and the atomic polarization.

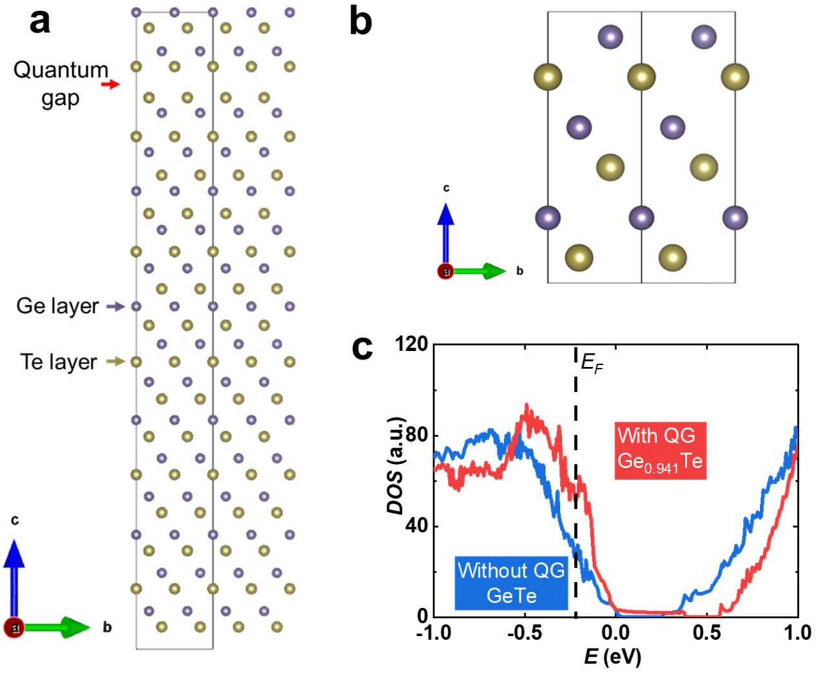


**Supplementary Fig. 9. The structural models for DFT calculations and electronic DOS of GeTe with QG ($Ge_{0.941}Te$) and without QG (GeTe). a**, The structural model of $Ge_{0.941}Te$. **b**, The structural model of GeTe. The primitive cells in a and b are encircled by solid lines. **c**, Electronic DOS of $Ge_{0.941}Te$ and GeTe.

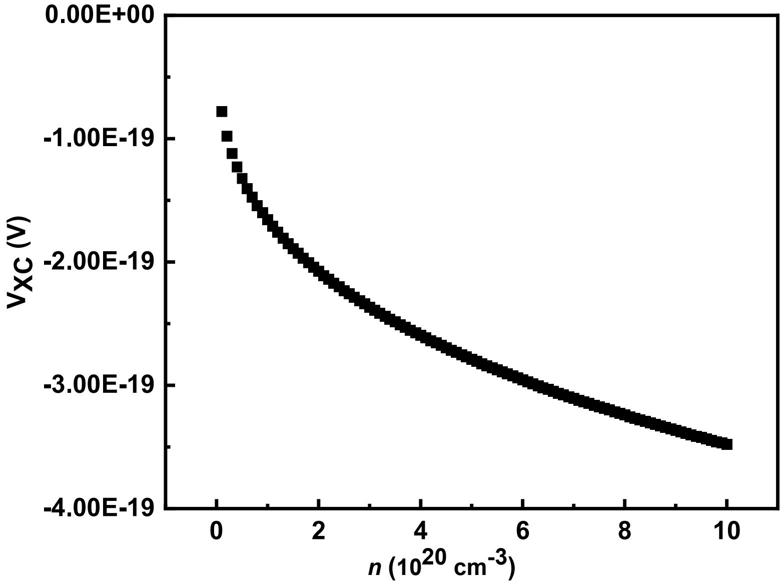


**Supplementary Fig. 10. The $V_{\mathrm{xc}}$ dependence on carrier concentration.**

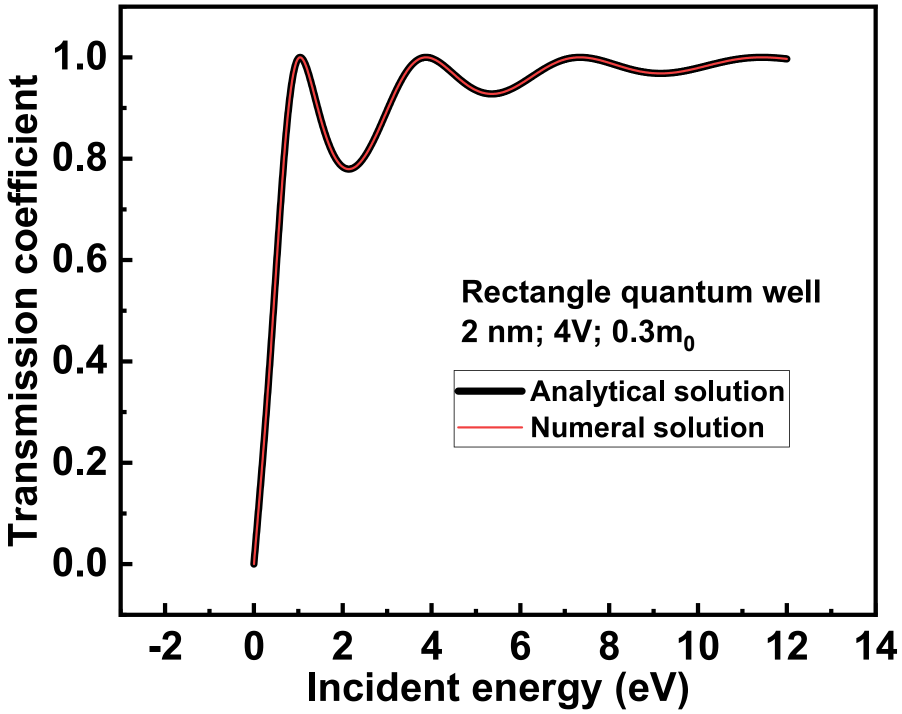


**Supplementary Fig. 11. The transmission coefficient calculated by the numerical method in this work compared with the standard analytical method.** The potential well is in the rectangular shape with its width=2 nm and depth=4 V. The effective mass is set to be $0.3m_0$. The potential energy of carriers is relative to the potential energy of the bottom of potential well.

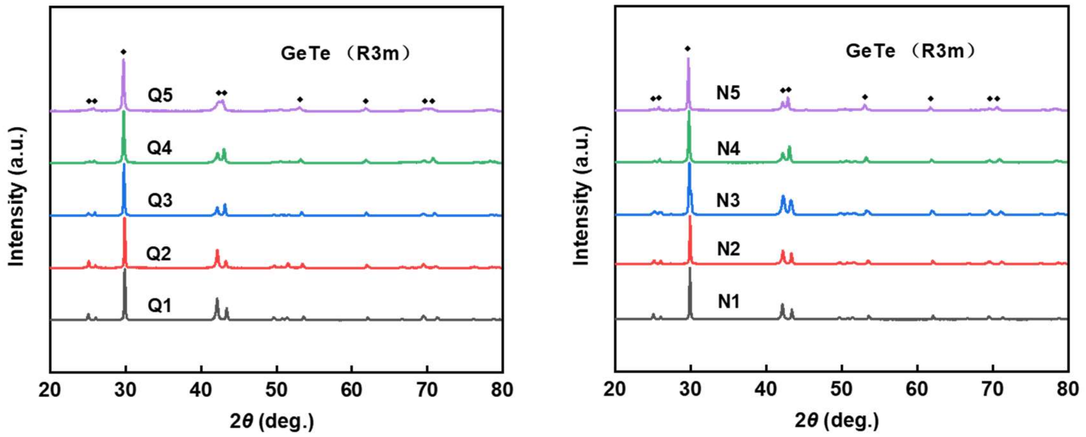


**Supplementary Fig. 12. X-ray diffraction (XRD) patterns of Q and N group samples.**

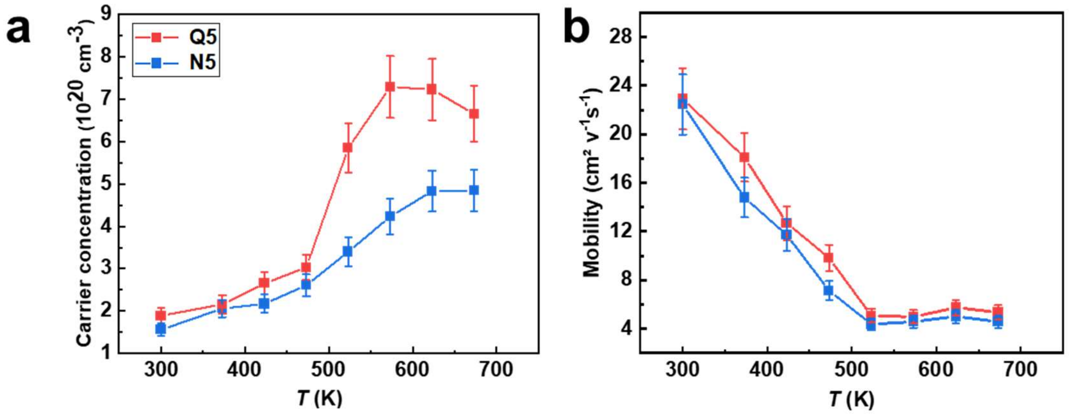


**Supplementary Fig. 13. The temperature-dependent carrier concentration and mobility of Q5 and N5 samples. a**, Carrier concentration. **b,** Mobility.

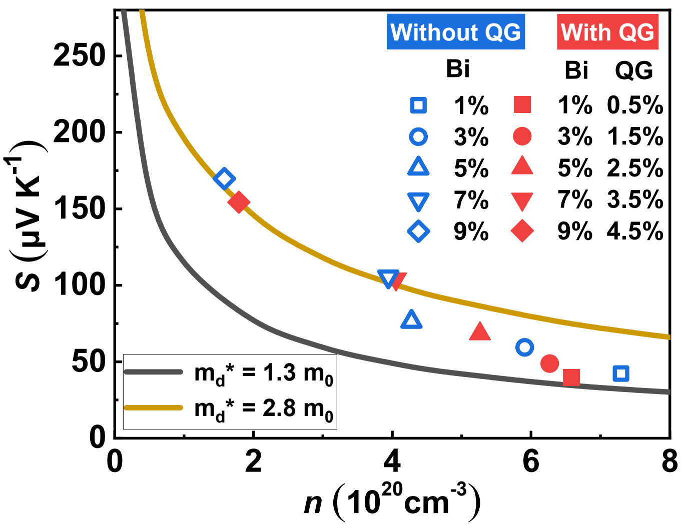


**Supplementary Fig. 14. The Pisarenko relationship of Q and N group samples.**

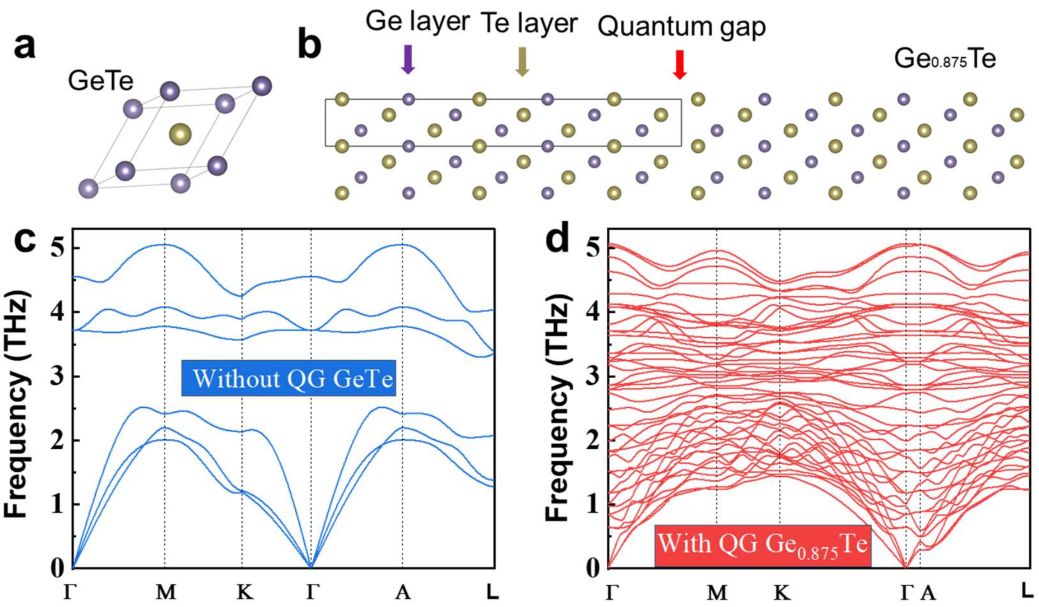


**Supplementary Fig. 15. The structural models for molecular dynamics simulations and phonon dispersions of GeTe without QG (GeTe) and GeTe with QG ($Ge_{0.875}Te$). a**, The structural model of GeTe. **b**, The structural model of $Ge_{0.875}Te$. The primitive cells in a and b are encircled by solid lines. **c**, The phonon dispersions of GeTe. **d**, The phonon dispersions of $Ge_{0.875}Te$.

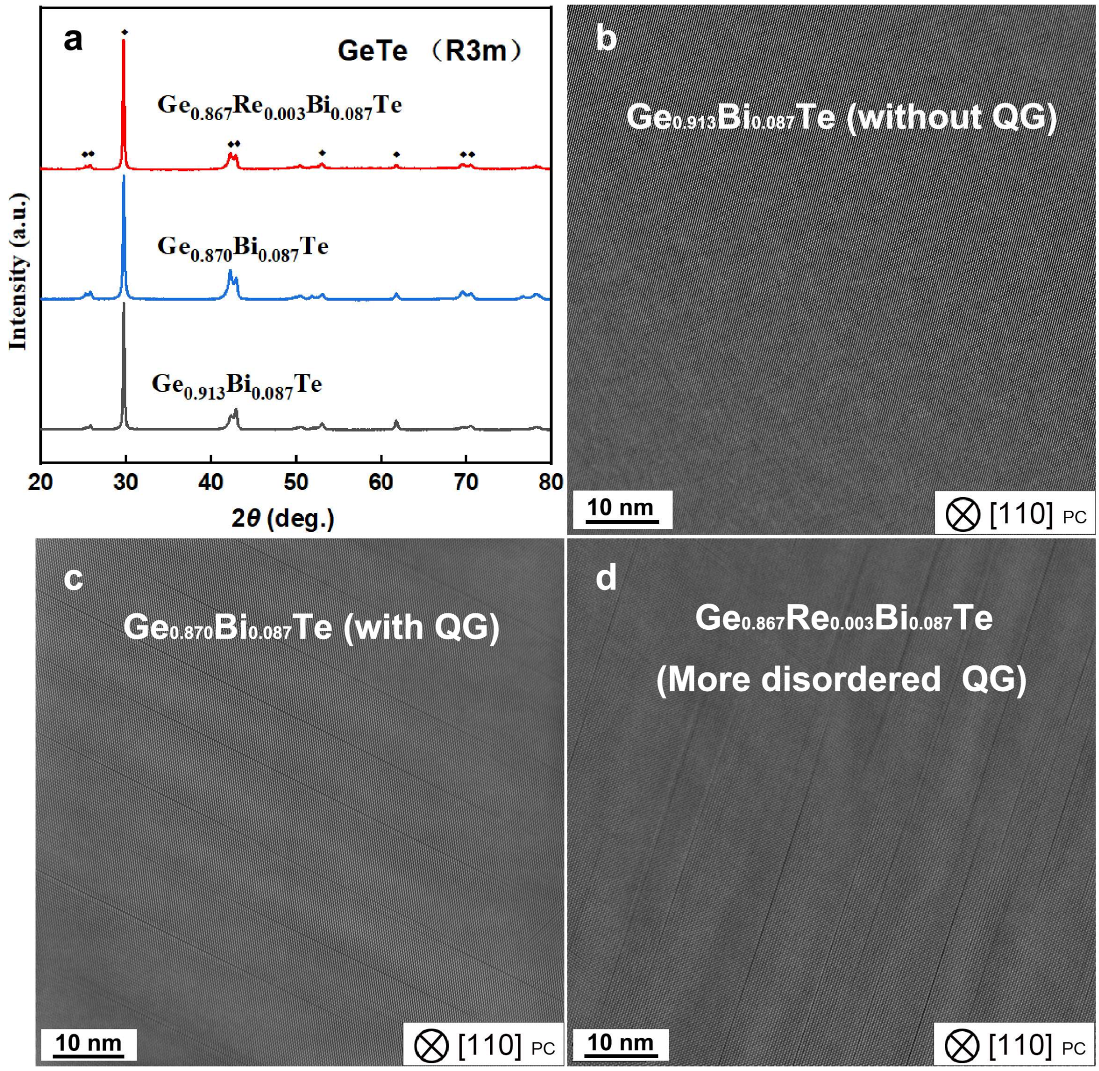


**Supplementary Fig. 16. Structure characterizations of $Ge_{0.913}Bi_{0.087}Te$ (without QG), $Ge_{0.870}Bi_{0.087}Te$ (with QG), and $Ge_{0.867}Re_{0.003}Bi_{0.087}Te$ (more disordered QG) for comparison. a,** XRD. **b,** The HAADF image of $Ge_{0.913}Bi_{0.087}Te$. **c,** The HAADF image of $Ge_{0.870}Bi_{0.087}Te$. **d,** The HAADF image of $Ge_{0.867}Re_{0.003}Bi_{0.087}Te$.

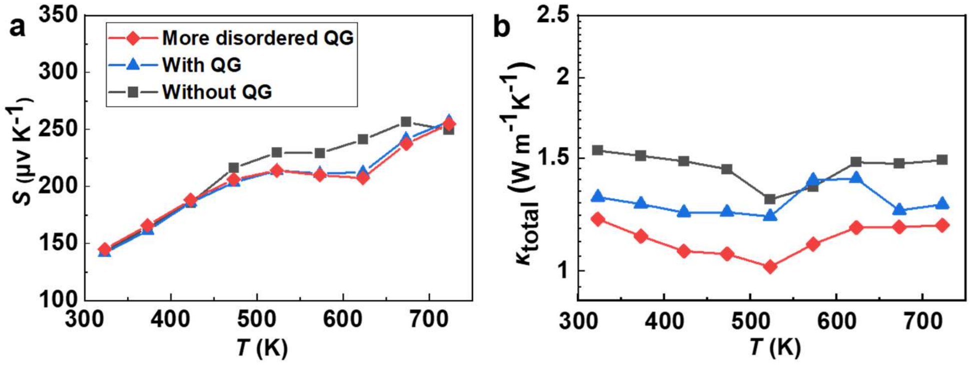


**Supplementary Fig. 17. Seebeck coefficients and total thermal conductivity of $Ge_{0.913}Bi_{0.087}Te$ (without QG), $Ge_{0.870}Bi_{0.087}Te$ (with QG), and $Ge_{0.867}Re_{0.003}Bi_{0.087}Te$ (more disordered QG).** **a**, Seebeck coefficients. **b**, total thermal conductivity. The uncertainty for $S$ (5%) and $\kappa_{total}$ (7%) are omitted for readability.

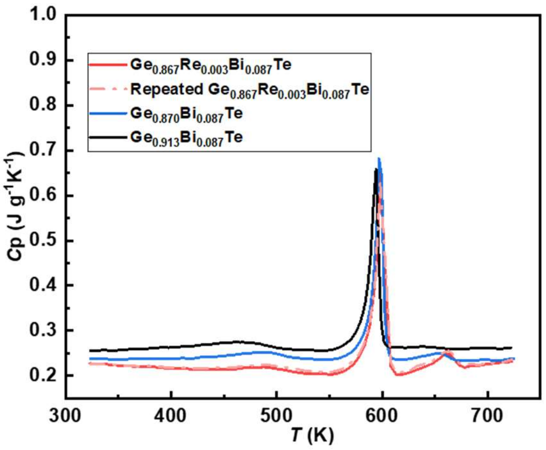


**Supplementary Fig. 18. Measured C*p* values of $Ge_{0.913}Bi_{0.087}Te$ (without QG), $Ge_{0.870}Bi_{0.087}Te$ (with QG), and $Ge_{0.867}Re_{0.003}Bi_{0.087}Te$ (more disordered QG).**

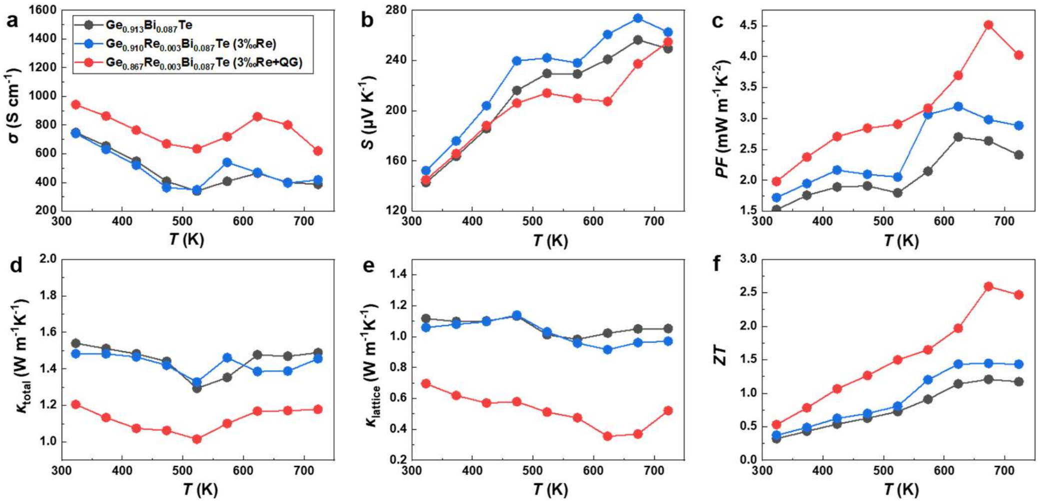


**Supplementary Fig. 19. The effect of Re and QG on thermoelectric properties. a**, The electrical conductivity. **b**, The Seebeck coefficient. **c**, The power factor. **d**, The total thermal conductivity based on measured thermal capacity. **e**, The lattice thermal conductivity based on measured thermal capacity. **f**, The figure of merit *ZT*. The uncertainty for $\kappa_{\mathrm{lattice}}$ (7%), $\sigma$ (5%), *S* (5%), *PF* (11%) and *ZT* (13%) are omitted for readability.

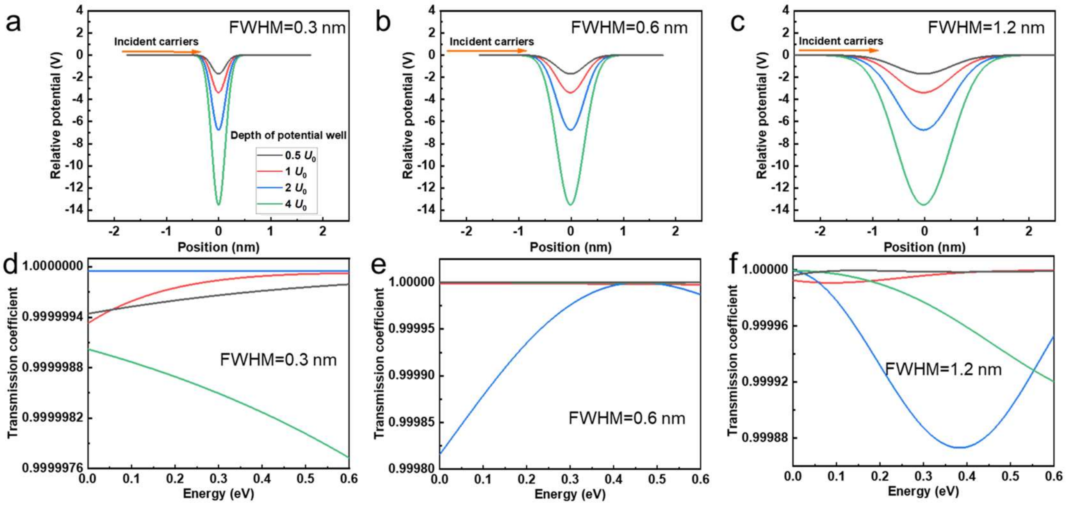


**Supplementary Fig. 20. The Gaussian-shaped potential wells with varied depth and width and the corresponding transmission coefficients. a**, A series of Gaussian-shaped potential wells with full width of half maximum (FWHM) of 0.3 nm. **b**, A series of Gaussian-shaped potential wells with FWHM of 0.6 nm. **c**, A series of Gaussian-shaped potential wells with FWHM of 1.2 nm. The value of $U_0$=3.3 V is the depth of the potential well of QG in Ge-Bi-Te alloy. **d**, **e**, **f**, The corresponding transmission coefficients of the potential wells shown in **a**, **b**, **c**, respectively. The effective mass is set to be $3m_0$, where $m_0$ is the rest mass of the electron. This is because $3m_0$ is closed to the density-states-effective mass of $Ge_{0.867}Re_{0.003}Bi_{0.087}Te$. It should be noted that our calculation results show that the transmission coefficient is not sensitive to the effective mass. The incident energy of carriers is relative to the energy of the plateau of potential well, which is the base energy of host material.

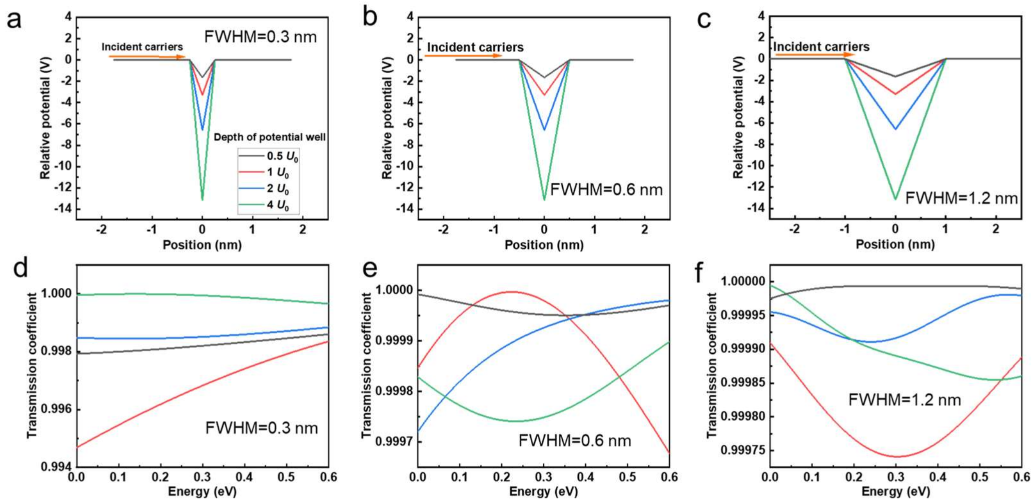


**Supplementary Fig. 21. The wedge-shaped potential wells with varied depth and width and the corresponding transmission coefficients.** **a**, A series of wedge-shaped potential wells with FWHM of 0.3 nm. **b**, A series of wedge-shaped potential wells with FWHM of 0.6 nm. **c**, A series of potential wells with FWHM of 1.2 nm. The value of $U_0$=3.3 V is the depth of the potential well of QG in Ge-Bi-Te alloy. **d**, **e**, **f**, The corresponding transmission coefficients of the potential wells shown in **a**, **b**, **c**, respectively. The effective mass is set to be $3m_0$, where $m_0$ is the rest mass of the electron. The incident energy of carriers is relative to the energy of the plateau of the potential well, which is the base energy of the host material.

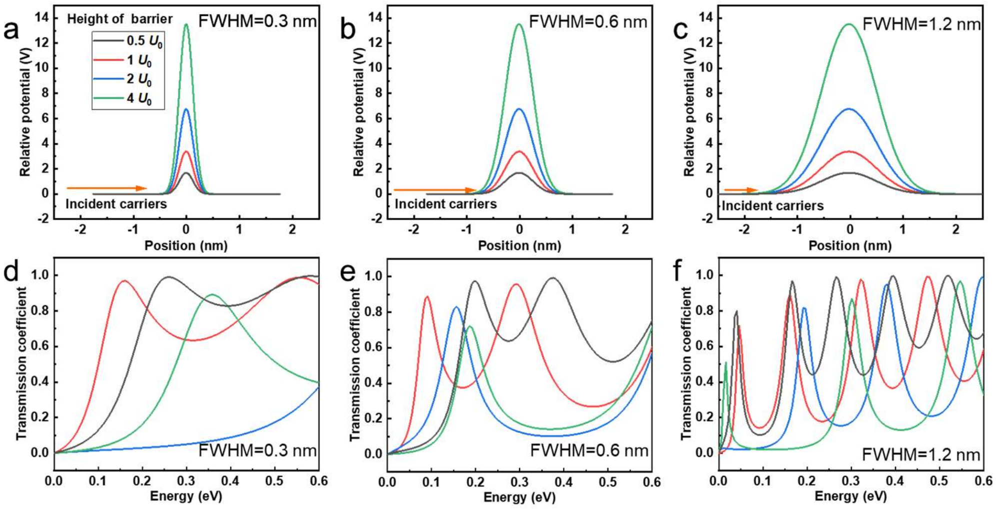


**Supplementary Fig. 22. The Gaussian-shaped potential barriers with varied depth and width and the corresponding transmission coefficients. a**, A series of Gaussian-shaped potential barriers with FWHM of 0.3 nm. **b**, A series of Gaussian-shaped potential barriers with FWHM of 0.6 nm. **c**, A series of Gaussian-shaped potential barriers of FWHM of 1.2 nm. The value of $U_0$= 3.3 V is the depth of the potential well of QG in Ge-Bi-Te alloy. **d**, **e**, **f**, The corresponding transmission coefficients of the potential wells shown in **a**, **b**, **c**, respectively. The effective mass is set to be $3m_0$, where $m_0$ is the rest mass of the electron. The incident energy of carriers is relative to the energy of the plateau of the potential well, which is the base energy of host material. These potential barriers can lead to the energy filter effect because low energy charge carriers have less transmission coefficient in contrast to high energy charge carriers.

**Supplementary Tables**

**Supplementary Table 1 The compositions of Q and N group samples**

| N group without QG | | | Q group with QG | | |
|---|---|---|---|---|---|
| Bi content | Label | Composition | Label | Composition | QG content |
| 1.0% | N1 | $Ge_{0.990}Bi_{0.010}Te$ | Q1 | $Ge_{0.985}Bi_{0.010}Te$ | 0.5% |
| 3.0% | N2 | $Ge_{0.970}Bi_{0.030}Te$ | Q2 | $Ge_{0.955}Bi_{0.030}Te$ | 1.5% |
| 5.0% | N3 | $Ge_{0.950}Bi_{0.050}Te$ | Q3 | $Ge_{0.925}Bi_{0.050}Te$ | 2.5% |
| 7.0% | N4 | $Ge_{0.930}Bi_{0.070}Te$ | Q4 | $Ge_{0.893}Bi_{0.070}Te$ | 3.5% |
| 9.0% | N5 | $Ge_{0.910}Bi_{0.090}Te$ | Q5 | $Ge_{0.864}Bi_{0.090}Te$ | 4.5% |

**Supplementary Table 2 Repeated carrier concentrations and mobilities**

| Sample | $n_1$ ($10^{20}$ cm$^{-3}$) | $\mu_1$ (cm$^2$ V$^{-1}$s$^{-1}$) | $n_2$ ($10^{20}$ cm$^{-3}$) | $\mu_2$ (cm$^2$ V$^{-1}$s$^{-1}$) | $n_3$ ($10^{20}$ cm$^{-3}$) | $\mu_3$ (cm$^2$ V$^{-1}$s$^{-1}$) |
|---|---|---|---|---|---|---|
| N1 | 6.26 | 58.89 | 7.54 | 48.90 | 8.09 | 45.57 |
| N2 | 5.03 | 51.70 | 6.24 | 41.68 | 6.46 | 40.26 |
| N3 | 4.13 | 43.50 | 3.70 | 48.55 | 4.43 | 40.55 |
| N4 | 3.54 | 28.43 | 3.92 | 25.67 | 4.37 | 23.03 |
| N5 | 1.56 | 22.45 | 1.50 | 23.35 | 1.68 | 20.84 |
| Q1 | 6.47 | 57.98 | 6.26 | 59.92 | 7.01 | 53.52 |
| Q2 | 5.69 | 49.89 | 6.21 | 45.71 | 6.91 | 41.08 |
| Q3 | 4.76 | 44.04 | 5.38 | 38.97 | 5.65 | 37.10 |
| Q4 | 3.28 | 29.43 | 3.78 | 25.54 | 5.10 | 18.93 |
| Q5 | 1.89 | 22.91 | 1.65 | 26.24 | 1.83 | 23.66 |

**Supplementary Table 3 Sound velocities (longitudinal sound velocity $v_l$, transverse sound velocity $v_t$, average sound velocity $v_a$) of Q and N group samples.** The poisson ratio r and the Grüneisen parameter $\gamma$ are derived based on the measured sound velocity.

| Sample | $v_l$ ($ms^{-1}$) | $v_t$ ($ms^{-1}$) | $v_a$ ($ms^{-1}$) | $\boldsymbol{r}$ | $\boldsymbol{\gamma}$ |
|---|---|---|---|---|---|
| N1 | 3500 | 1991 | 2213 | 0.2608 | 1.553 |
| N2 | 3428 | 1957 | 2174 | 0.2583 | 1.541 |
| N3 | 3393 | 1940 | 2155 | 0.2571 | 1.535 |
| N4 | 3355 | 1911 | 2124 | 0.2597 | 1.548 |
| N5 | 3296 | 1867 | 2076 | 0.2637 | 1.568 |
| Q1 | 3507 | 1971 | 2193 | 0.2691 | 1.596 |
| Q2 | 3460 | 1941 | 2159 | 0.2704 | 1.603 |
| Q3 | 3431 | 1895 | 2111 | 0.2787 | 1.658 |
| Q4 | 3393 | 1863 | 2077 | 0.2842 | 1.679 |
| Q5 | 3282 | 1780 | 2006 | 0.2850 | 1.683 |

**Supplementary Table 4 The compositions of samples for *ZT* enhancement.**

| Label | Composition | Bi content | QG content |
|---|---|---|---|
| Without QG | $Ge_{0.913}Bi_{0.087}Te$ | 8.7% | 0% |
| With QG | $Ge_{0.870}Bi_{0.087}Te$ | 8.7% | 4.4% |
| More disordered QG | $Ge_{0.867}Re_{0.003}Bi_{0.087}Te$ | 8.7% | 4.4% |

**Supplementary Table 5 The longitudinal sound velocity $v_l$, transverse sound velocity $v_t$, average sound velocity $v_a$, the poisson ratio $r$ and the Grüneisen parameter of samples for *ZT* enhancement.** The compositions for GeTe without QG, with QG, and more disordered QG are $Ge_{0.913}Bi_{0.087}Te$, $Ge_{0.870}Bi_{0.087}Te$, and $Ge_{0.867}Re_{0.003}Bi_{0.087}Te$, respectively.

| Label | $v_l$ (ms$^{-1}$) | $v_t$ (ms$^{-1}$) | $v_a$ (ms$^{-1}$) | $r$ | $\gamma$ |
|---|---|---|---|---|---|
| **Without QG** | 3350 | 1902 | 2114 | 0.2623 | 1.561 |
| **With QG** | 3287 | 1807 | 2014 | 0.2837 | 1.676 |
| **More disordered QG** | 3298 | 1774 | 1980 | 0.2965 | 1.751 |

**References**


1. Frolov, A. S. et al. Atomic and Electronic Structure of a Multidomain GeTe Crystal. *ACS Nano* **14**, 16576–16589 (2020).
2. Kasap, K. O. Principles of Electronic Materials and Devices. *McGraw-Hill* (2006).
3. Littlewood, P. B. The dielectric constant of cubic IV-VI compounds. *J. Phys. C Solid State Phys.* **12**, 4459–4468 (1979).
4. Wang, R. et al. Formation of resonant bonding during growth of ultrathin gete films. *NPG Asia Mater.* **9**, (2017).
5. Şenadim Tüzemen, E., Turkoglu, A., Ergun, Y., Sokmen, I. & Tanatar, B. Effect of exchange-correlation potential on the plasmon dispersions in a doped symmetrical double quantum well. *Phys. Status Solidi Basic Res.* **244**, 635–641 (2007).
6. Datta, S. & Houten, H. V. Electronic Transport in Mesoscopic Systems. *Phys. Today* **49**, (1996).
7. Lewenkopf, C. H. & Mucciolo, E. R. The recursive Green's function method for graphene. *J. Comput. Electron.* **12**, 203–231 (2013).
8. Chen, R. et al. Using nonlocal surface transport to identify the axion insulator. *Phys. Rev. B* **103**, L241409 (2021).
9. Chen, R., Wang, C. M., Liu, T., Lu, H.-Z. & Xie, X. C. Quantum Hall effect originated from helical edge states in Cd 3 As 2. *Phys. Rev. Res.* **3**, 33227 (2021).
10. Chen, R., Liu, T., Wang, C. M., Lu, H.-Z. & Xie, X. C. Field-tunable one-sided higher-order topological hinge states in Dirac semimetals. *Phys. Rev. Lett.* **127**, 66801 (2021).
11. Li, W., Carrete, J., Katcho, N. A. & Mingo, N. ShengBTE: A solver of the Boltzmann transport equation for phonons. *Comput. Phys. Commun.* **185**, 1747–1758 (2014).
12. Kurosaki, K., Kosuga, A., Muta, H., Uno, M. & Yamanaka, S. $Ag_9TlTe_5$: A

high-performance thermoelectric bulk material with extremely low thermal conductivity. *Appl. Phys. Lett.* **87**, 1–4 (2005).

13. Jia, T., Chen, G. & Zhang, Y. Lattice thermal conductivity evaluated using elastic properties. *Phys. Rev. B* **95**, 1–6 (2017).
14. Liu, Z. et al. High Power Factor and Enhanced Thermoelectric Performance in Sc and Bi Codoped GeTe: Insights into the Hidden Role of Rhombohedral Distortion Degree. *Adv. Energy Mater.* **10**, 2002588 (2020).
15. Shuai, J., Sun, Y., Tan, X. & Mori, T. Manipulating the Ge Vacancies and Ge Precipitates through Cr Doping for Realizing the High-Performance GeTe Thermoelectric Material. *Small* **16**, 1906921 (2020).
16. Lee, H. *Thermoelectrics: design and materials*. (John Wiley & Sons, 2016).
17. Clementi, E., Raimondi, D. L. & Reinhardt, W. P. Atomic screening constants from SCF functions. II. Atoms with 37 to 86 electrons. *J. Chem. Phys.* **47**, 1300–1307 (1967).
